# Identifying children at risk in Swedish Child Health Services

**DOI:** 10.1177/14034948241277862

**Published:** 2024-09-25

**Authors:** Mattias Wennergren, Anna Fäldt

**Affiliations:** 1 General Practice, Family Medicine, School of Public Health and Community Medicine, Institute of Medicine, Sahlgrenska Academy, University of Gothenburg, Sweden; 2 Research, Education, Development & Innovation, Primary Health Care, Region Västra Götaland, Göteborg, Sweden; 3 Region Västra Götaland, Department of Child Health Services, Göteborg, Sweden; 4 Child Health and Parenting (CHAP), Department of Public Health and Caring Sciences, Uppsala University, Uppsala, Sweden

**Keywords:** Child Health Services, socioeconomic factors, social medicine, socioeconomic disparities in health, Sweden

## Abstract

**Background:**

Child Health Services plays an important role in identifying at-risk children and intervening early to break negative trends in child health. Sociodemographic risk factors can impact the workload of Child Health Services and affect the possibilities of providing the national child healthcare programme.

**Aims:**

This study aims to present the sociodemographic characteristics of families who are registered within the Child Health Services, as defined by the Child adjusted Care Need Index.

**Methods:**

By collecting personal identification numbers from children six years or younger registered at a child healthcare centre, and combining this with their caregiver’s sociodemographic background, this study was able to create a sociodemographic index for each child healthcare centre in Sweden.

**Results:**

The study included 687,543 children and 1,335,540 caregivers from 981 child healthcare centres in Sweden. Approximately 21% of all children in the study population had a caregiver born in Southern or Eastern Europe outside the European Union, or in Africa, Asia, or South America, 7% had single parents, 17% had at least one unemployed caregiver, and 9% had at least one caregiver who had not completed high school. The average input values and the average index values varied widely both between and within the regions.

**Conclusions:**

**This study displays a large variation in sociodemographics for child healthcare centres both within and between regions. Since several regions and national agencies in Sweden use the Child adjusted Care Need Index, it is necessary to keep the dispersion in mind.**

## Background

The Swedish Child Health Services is an integral and highly reputable part of the national public healthcare system. Most children participate in regular visits to the Child Health Services, at a Child Healthcare Centre (hereafter centre), with the attendance being almost 100% [1]. The Child Health Services aims to improve children’s physical, psychological and social well-being by promoting health and development, preventing illnesses and detecting emerging problems in their lives. Within the Child Health Services framework, families receive preventative healthcare services such as vaccinations, parental support and healthy lifestyle promotion [2]. The universal aspects of the national programme are accessible to all children, with additional visits or referrals available as needed [2,3]. These services adhere to a national programme divided into three parts: universal, targeted and indicated interventions. The interventions are based on the concept of proportionate universalism, and the level of intervention provided is tailored to meet the specific needs of each family [2,4]. Although there is a national consensus to follow the national programme, the delivery of Child Health Services varies greatly between and within regions. For example, parts of the programme are not implemented in all regions, and the degree of provided services such as home visits and screenings varies [5,6].

The Child Health Services plays an important role in identifying at-risk children and intervening early to break negative trends in child health. Sociodemographic risk factors can impact the workload of Child Health Services and affect the possibilities of providing the national child healthcare programme. Several regions in Sweden use the Care Need Index for resource allocation; however, the effect of the allocation is not sufficiently evaluated. Child adjusting the Care Need Index is one way of calculating possible additional needs of Child Health Services. This study aims to describe the sociodemographic characteristics of families who are in contact with the centre, as defined by the variables included in the Child adjusted Care Need Index. It also analyses the disparities among the centres’ sociodemographic circumstances. The Care Need Index is a weighted relative index, based on General Practitioners’ assessments of sociodemographic groups that might influence the resources needed for primary healthcare, developed in the late 1990s [7,8]. Variables included in the Care Need Index are presented in Table I, and a high index value indicates an increased risk for additional healthcare needs. Today, the Care Need Index is a standard measurement from Statistics Sweden, used in several contexts when comparing probable healthcare needs among groups with diverse sociodemographic composition. In most Swedish regions, the Care Need Index is used to allocate resources, mainly financial, to healthcare providers. The purpose is to reduce disparities in healthcare access and health outcomes among different sociodemographic groups. In some regions, up to 21% of the public funding for primary healthcare is divided with the Care Need Index [9]. The resources should enable an increase in number of professionals and increase the number of visits at specific centres. Several regions also use the Care Need Index as a variable for analysing whether the annual production varies between sociodemographic settings within the Child Health Services, to enable the public health goal of equity in health [10].

**Table I.** Variables included in the Care Need Index.

Variable	Relative Care Need Index weight	Included in the Child adjusted Care Need Index
Being over 65 years and living alone	6.15	No
Being 16–64 years old and unemployed	5.13	Yes^a^
Being born in Southern or Eastern Europe outside the European Union, or in Africa, Asia or South America	5.72	Yes^a^
Being a single parent with children under 17 years	4.19	Yes^a^
Being 25–64 years old and having at most nine years of compulsory schooling	3.97	Yes^a^
Being under five years of age	3.23	No
Being above one year of age and having recently moved across parish borders	4.19	Yes^a^

a Based on caregivers of children registered at each centre.

New innovations and task and demographic shifts over the past two decades could influence how healthcare utilisation and needs differ between sociodemographic contexts. Since the Care Need Index was developed, it has not changed; thus, it could be challenging to apply it in the changing healthcare setting. Previous research has found a weak correlation between increased primary healthcare visits and increased Care Need Index value [9]. This could imply that other factors beyond the sociodemographics included in the Care Need Index might affect healthcare utilisation [11].

### Child adjusted Care Need Index

Some studies conclude that there are differences in attendance and access to healthcare among families from different sociodemographic backgrounds and, thereby, risks of disparities in health outcomes [12,13]. With the aim to better reflect the sociodemographic risk for increased needs among families from the Child Health Services, several regions have previously adjusted the Care Need Index to a specific Child adjusted Care Need Index. This index is based on the caregivers of registered children (0–6 years old) at the centres and excluded two redundant sociodemographic variables (Table I).

Resource allocation within the Child Health Services based on sociodemographic variables has proven challenging to implement, failing to reduce differences in healthcare utilisation between groups [14]. Even though several regions use the Child adjusted Care Need Index for resource allocation and to analyse differences [6,14] it is not validated or evidence-based. The Swedish national child health service quality register [5] included the Child adjusted Care Need Index in 2022. Several participating regions in Sweden requested this development to enable the analysis of Child Health Services-related data combined with data on sociodemographic contexts.

To our knowledge, this is the first attempt to explore similarities and disparities among the sociodemographic backgrounds, as defined by the variables included in the Child adjusted Care Need Index, of children registered at centres within the context of Child Health Services.

## Methods

### Study population and variables

This study included data from 20 out of Sweden’s 21 regions encompassing 688,190 children and 1,336,697 caregivers from 1013 centres. To avoid bias from skewed populations at small centres, the centres that had fewer than 50 registered children were excluded; consequently, a new national index was calculated for 981 centres. The final number of children included in this study was 687,543 and there was a total of 1,335,540 caregivers. The proportion of publicly or privately operated centres is presented in Table II, and Table III presents the total study population and characteristics per region and average percentages of sociodemographic variables.

**Table II.** Number of child healthcare centres per region and type of operation. Note that all centres in this context are publicly financed. *n* (%).

Region	Private	Public	Missing values
Blekinge	6 (33%)	12 (67%)	
Dalarna	5 (18%)	23 (82%)	
Gotland	0 (0%)	3 (100%)	
Gävleborg	10 (30%)	23 (70%)	
Halland	23 (49%)	24 (51%)	
Jämtland	5 (19%)	21 (81%)	
Jönköping	0 (0%)	25 (100%)	
Kalmar	11 (30%)	26 (70%)	
Kronoberg	11 (34%)	21 (66%)	
Norrbotten	5 (18%)	23 (82%)	
Skåne	69 (46%)	79 (53%)	2 (1%)
Stockholm	64 (55%)	52 (44%)	1 (1%)
Södermanland	9 (31%)	20 (69%)	
Uppsala	17 (39%)	27 (61%)	
Västra Götaland	101 (49%)	104 (51%)	
Värmland	3 (12%)	22 (88%)	
Västernorrland	12 (39%)	19 (61%)	
Västmanland	16 (55%)	12 (41%)	1 (3%)
Örebro	5 (17%)	24 (83%)	
Östergötland	11 (24%)	34 (76%)	
**Total**	383 (39%)	594 (61%)	4 (0%)

**Table III.** Mean value among all child healthcare centres per region for Care Need Index input value.

Average per centre in every region					
	Caregivers of children under six years old				Children
Region	Born in Southern or Eastern Europe outside the European Union, or in Africa, Asia or South America	Single parents	Unemployed	Having at most nine years of compulsory schooling	Above one year of age, having recently moved across parish borders
**Blekinge**	18%	6%	21%	10%	10%
**Dalarna**	16%	8%	17%	12%	8%
**Gotland**	7%	7%	14%	7%	12%
**Gävleborg**	22%	9%	25%	13%	11%
**Halland**	11%	5%	13%	7%	15%
**Jämtland**	11%	7%	15%	8%	10%
**Jönköping**	19%	5%	16%	10%	11%
**Kalmar**	16%	6%	17%	10%	12%
**Kronoberg**	21%	6%	21%	11%	11%
**Norrbotten**	11%	6%	14%	8%	8%
**Skåne**	20%	7%	21%	10%	14%
**Stockholm**	23%	7%	14%	7%	16%
**Södermanland**	27%	9%	23%	14%	14%
**Uppsala**	18%	6%	15%	8%	14%
**Värmland**	13%	7%	18%	9%	9%
**Västernorrland**	15%	7%	20%	10%	11%
**Västra Götaland**	20%	7%	17%	9%	14%
**Västmanland**	28%	9%	22%	13%	13%
**Örebro**	22%	7%	22%	12%	11%
**Östergötland**	18%	7%	18%	10%	14%
**Average for all centres in the study**	19%	7%	18%	10%	13%

The centres’ addresses were used to map their respective locations (urban, suburban or rural), based on the Swedish Association of Municipalities and Regions’ classification of municipalities [15].

From the calculations of the Care Need Index this study use both the calculated index value as well as the input data from the sociodemographic categories [7–9]. The descriptive statistics were summarised and explored using Microsoft Excel and IBM Statistical Packages for the Social Sciences (SPSS v. 28.0.1).

### The calculations of a Child adjusted Care Need Index

Twenty out of the 21 regions in Sweden issued a personal identification number to all children born between 1 July 2016 and 30 June 2022 (except for region Stockholm, which could include only children born between 1 January 2017 and 31 October 2022), registered at a centre in August 2022. Statistics Sweden, thereafter, linked the personal identification number for each child to sociodemographic information regarding the child’s caregivers since the child him or herself is not directly factored into the index. Statistics Sweden calculated a Child adjusted Care Need Index for each centre. These calculations produced a weighted index per region and nationally, grouped by centre.

To use the Child adjusted Care Need Index value on a national level the given population refers to the median nationally. Therefore, this study describes the distribution of index values from all included centres both regionally and nationally.

## Results

The study included data from 981 centres and a total of 687,543 children and 1,335,540 caregivers in Sweden. Among all the centres included in this study, approximately 61% were publicly operated, with variations between 41% and 100% across the different regions (Table II).

The 981 centres were distributed diversely across Sweden and the centres were not evenly distributed across the three categories at a national level. Some regions did not have centres in smaller towns or rural areas and others had over 90% of all centres in rural areas. Many centres (44%) were located in medium-sized towns or smaller towns (see Supplementary material 1 online.

Approximately 21% of all the children in the study population had a caregiver born in Southern or Eastern Europe outside the European Union, or in Africa, Asia, or South America (hereafter immigrant families), 7% had a single parent, 17% had at least one caregiver who was unemployed and 9% had at least one caregiver who had not completed high school (Table III). The average values per centre vary widely both between and within the regions. Between regions the results display a difference of between 7% and 28% in percentage of children in immigrant families. The unemployment rate of caregivers differed between 13% and 25% between regions. Within regions the variation between centres was extreme. The range of children in immigrant families differed as much as between 4% and 82%. The within regional variation in unemployment rate for caregivers differed between 4% and 62% (see Supplementary material 2). However, Table III shows only the comparison between the regions’ average values. For all sociodemographic characteristics per centre see Supplementary material 2.

**Figure 1.**
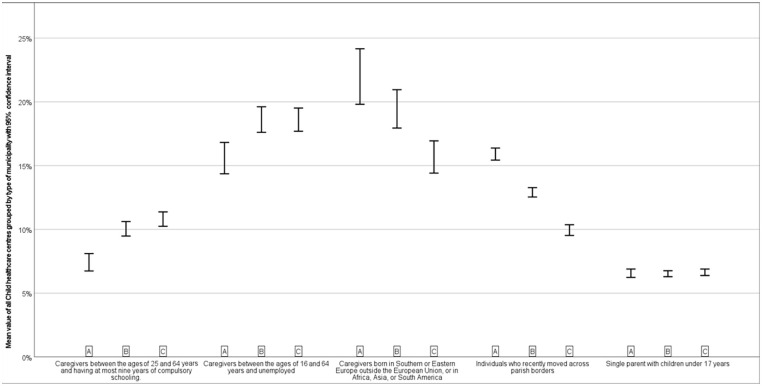
Mean value for all child healthcare centres in the three groups of municipalities with 95% confidence interval. A stands for large cities and municipalities near large cities, B for medium-sized towns and municipalities near medium-sized towns and C for smaller towns/urban areas and rural municipalities. Variables are based on caregivers of children under six years old.

The three groups of municipalities have varying percentages of registered immigrant families, unemployment rates, families that move and low educational level (Figure 1). The only factor that does not differ across geographical levels is the prevalence of single parents.

The index values differ from the regional to the national context, highlighting differences between the national and regional Child adjusted Care Need Index (Table IV). Most regions have small differences between their national and regional values, but some show larger differences. The centres’ values can shift either positively or negatively depending on the index’s context. In this case, the regional value is compared solely with the median within that region. However, in the national context, the values are compared with the median for the entire nation. For example, in region Sörmland the mean difference between national and regional Care Need Index value was 0.5, which means that the centres are on average more deprived than in the national context. The opposite pattern is revealed by region Halland, where the centres on average are less deprived in a national context than in the regional context.

**Table IV.** Average differences between regional and national Child adjusted Care Need Index per region. A positive value indicates that the centres have a higher Care Need Index value when comparing nationally than within the region.

Region	Mean difference between national and regional Care Need Index value
Blekinge	0.1
Dalarna	–0.1
Gotland	–0.2
Gävleborg	0.3
Halland	–0.3
Jämtland	–0.2
Jönköping	0.0
Kalmar	0.0
Kronoberg	0.0
Norrbotten	–0.2
Skåne	0.1
Stockholm	–0.1
Södermanland	0.5
Uppsala	0.0
Västra Götaland	0.0
Värmland	–0.1
Västernorrland	0.0
Västmanland	0.4
Örebro	0.3
Östergötland	–0.1

Among the 6% of centres with a Child adjusted Care Need Index at 2.5 or higher, the average percentage of immigrants is 63%. In contrast, for centres with 0.5 or below Child adjusted Care Need Index (bottom 6%), the average of immigrants percentage is 4% (see Supplementary material 2). The corresponding numbers for the unemployment rate are 43% and 7%, respectively. These differences between extremes are large and influenced by the heavy weights in the index equations and the variability across centres.

## Discussion

The distribution of centres in urban or rural environments varies across regions, as do the socioeconomic burdens. These differences in demographic and geographical conditions are wide and could pose challenges in creating consistent healthcare programmes for children nationwide [6,16]. The study shows disparities in sociodemographic circumstances across areas where the same healthcare programme is supposed to be applied. These differences can affect the degree to which the programme is delivered as well as the quality of the services provided; however, this is just a conjecture. Several reports from Sweden highlight difficulties in delivering the universal aspect of the healthcare programme in both rural and urban settings [5,6,14]. In rural settings, challenges emerge when dispersed geographical context is combined with lower levels of education and higher rates of unemployment within the population. In contrast, urban settings have denser populations combined with challenges arising from higher levels of movement and immigration. This relationship is present at a group level; the Child adjusted Care Need Index does not differentiate between individual factors for families or whether they accumulate or whether they co-vary with each other.

Since the Child adjusted Care Need Index does not account for factors such as being over 65 years old and living alone, the weights assigned to immigrants and unemployment have become very high. As immigrants in Sweden are not evenly settled across the country, the urban areas surrounding major cities are overrepresented at the high-index end. The differences between the proportion of immigrants, proportion of unemployment and the educational levels between and within regions are very high within the general population [17,18]. Hence, it could be relevant to evaluate its effects in the sociodemographic environment if other social, economic or demographic factors could be more relevant or if the use of the Care Need Index or Child adjusted Care Need Index should increase. Therefore, we suggest further investigation into the regional and local differences in socioeconomic deprivation and its effect on healthcare utilisation within the Child Health Services.

As highlighted above, there are several regions that differ in their regional context when comparing with the national context. These differences have an implication for resource allocation, where it is necessary to pay attention to the scale of comparison (differences between and within regions). This is especially important when allocating resources with the Child adjusted Care Need Index as part of a proportionate universalism approach, where the method for dividing resources can affect the outcome. Large differences between and within regions have also been reported by the Swedish National Board of Health and Welfare but have not been analysed on a centre level [6,14].

There is a possibility that access to healthcare and health outcomes can differ between centres with low and high index values. As several studies have concluded, the lack of access to healthcare among immigrants is a problem that can widen the health gap in society [19,20]. Similar results have been found when analysing low and high levels of education [12,19,21]. In addition there could be other factors influencing both access to and utilisation of healthcare, such as health literacy, language or trust. This study also shows that these factors can overlap, as Child adjusted Care Need Index values are often a combination of both economic and social deprivation. The sociodemographic differences in health and access to healthcare are discussed in several studies, editorials and reviews [12,14,19,22,23]. In the presence of more than one risk factor, the total risk may accumulate and increase [24]. Adjusting resources to enable proportionate universalism for equitable access to preventative services and early interventions can enhance overall child health and development. Regional and national authorities need to make sure that resources allocated in the Swedish Healthcare System, with the commendable goal of delivering proportionate universalism, are allocated based on the correct grounds.

It is unclear whether the programme is universally offered and whether there are differences in attendance between sociodemographic groups. Sociodemographic factors can affect the ability to provide the national programme and maintain universal access to Child Health Services while ensuring sufficient quality for the well-being of children. Allocating resources to enable equity in healthcare access is crucial for closing the health gap between and within regions and between sociodemographic groups [25,26]. Countless ways of calculating and adjusting payments for allocating resources for equity exist, and the Care Need Index and Child adjusted Care Need index are methods that are widespread in Sweden. However, our knowledge is limited in whether other countries use the variables included in the Care Need Index in any form for evaluating healthcare utilisation. In other countries there are challenges in both identifying which families are in need for extra support and allocating resources for these families [25,26]. Once adjusted to the national and local context in that country, the Care Need Index could be a part of resource allocation or identification of families in greater need in order to enable proportionate universalism.

### Limitations and strengths

The main strength of this study is that it covers an exploration of the Child adjusted Care Need Index, which to our knowledge is the first on a national level in Sweden. It also describes the dispersion of sociodemographic contexts encountered by centres across the nation. Another strength is the collection of data from 20 out of 21 regions, involving 1014 centres and over 1.3 million participants. When excluding small centres, the participation rate was very high, encompassing 687,543 children and a total of 1,335,540 caregivers.

However, this study has several limitations. First, the study does not analyse the dispersion of units within each regional category. Second, the data used in this study is not linked to healthcare utilisation or health status. Third, the study does not use individual-level data, which forms the basis for the index calculation.

## Conclusions

This study explores the distribution of the Child adjusted Care Need Index and reveals major differences between regional and national index values in certain Swedish regions. It also shows wide dispersion in index values and the sociodemographic background used in index calculation, both within and between regions. Since several regions and national agencies in Sweden use the Care Need Index or Child adjusted Care Need Index, it is necessary to keep the dispersion in mind.

We suggest further research to explore sociodemographic differences in healthcare access and to test whether other variables could be used for a revised child adjusted index that considers these differences. We also suggest studying how the data from national registries can be used to allocate resources to more deprived populations.

Finally, we suggest a more comprehensive evaluation and study of how sociodemographic factors within families affect healthcare needs and utilisation and how they interact with each other.

## Supplemental Material

sj-docx-1-sjp-10.1177_14034948241277862 – Supplemental material for Identifying children at risk in Swedish Child Health Services Supplemental material, sj-docx-1-sjp-10.1177_14034948241277862 for Identifying children at risk in Swedish Child Health Services by Mattias Wennergren and Anna Fäldt in Scandinavian Journal of Public Health
