# Supplementary material for: Identifying children at risk in Swedish Child Health Services
Source: Scand J Public Health. 2024 Sep 25;53(8):813–9. doi: 10.1177/14034948241277862 (PMC12619851; doi:10.1177/14034948241277862)
Supplement: sj-docx-1-sjp-10.1177_14034948241277862 – Supplemental material for Identifying children at risk in Swedish Child Health Services [file sj-docx-1-sjp-10.1177_14034948241277862.docx]

Supplementary material 1:

| Region (N/%)  Number of Child Healthcare Centres in each participating region divided by categories of municipalities | | | |
| --- | --- | --- | --- |
|  | A. Large cities and municipalities near large cities | B. Medium-sized towns and municipalities near medium-sized towns | C. Smaller towns/urban areas and rural municipalities |
| Blekinge |  | 2 (11%) | 16 (89%) |
| Dalarna |  | 6 (21%) | 22 (79%) |
| Gotland |  |  | 3 (100%) |
| Gävleborg |  | 17 (52%) | 16 (48%) |
| Halland | 11 (23%) | 21 (45%) | 15 (32%) |
| Jämtland |  | 14 (54%) | 12 (46%) |
| Jönköping |  | 15 (60%) | 10 (40%) |
| Kalmar |  | 17 (46%) | 20 (54%) |
| Kronoberg |  | 22 (69%) | 10 (31%) |
| Norrbotten |  | 12 (43%) | 16 (57%) |
| Skåne | 51 (34%) | 84 (56%) | 13 (9%) |
| Stockholm | 105 (90%) | 5 (4%) | 6 (5%) |
| Södermanland |  | 16 (55%) | 13 (45%) |
| Uppsala | 3 (7%) | 41 (93%) |  |
| Västra Götaland | 112 (55%) | 43 (21%) | 50 (24%) |
| Värmland |  | 15 (60%) | 10 (40%) |
| Västernorrland |  | 14 (45%) | 17 (55%) |
| Västmanland |  | 22 (76%) | 6 (21%) |
| Örebro |  | 23 (79%) | 6 (21%) |
| Östergötland |  | 41 (91%) | 4 (9%) |
| **Total** | 282 (29%) | 430 (44%) | 265 (27%) |

Supplementary material 2 **All Child healthcare centres in the study sorted by region and in descending order based on National Child adjusted Care Need Index-value with numbers for Regional Child adjusted Care Need Index and percentages of sociodemographic background characteristics.**

| Child Healthcare Centre | Region | National Child adjusted Care need index | Regional Child adjusted Care need index | Caregivers born in Southern or Eastern Europe outside the European Union, or in Africa, Asia, or South America | Single parent with children under 17 years | Individuals over the age of 1 recently moved across parish borders | Caregivers between the ages of 16–64 years and unemployed | Caregivers between the ages of 25–64 years and having at most nine years of compulsory schooling. |
| --- | --- | --- | --- | --- | --- | --- | --- | --- |
| 1 | Blekinge | 3.0 | 2.7 | 62% | 9% | 11% | 55% | 26% |
| 2 | Blekinge | 1.9 | 1.7 | 39% | 6% | 6% | 36% | 18% |
| 3 | Blekinge | 1.7 | 1.5 | 31% | 5% | 15% | 28% | 14% |
| 4 | Blekinge | 1.3 | 1.2 | 23% | 7% | 4% | 25% | 13% |
| 5 | Blekinge | 1.3 | 1.1 | 21% | 7% | 8% | 24% | 11% |
| 6 | Blekinge | 1.3 | 1.1 | 18% | 9% | 10% | 22% | 11% |
| 7 | Blekinge | 1.2 | 1.0 | 16% | 7% | 7% | 23% | 13% |
| 8 | Blekinge | 1.1 | 1.0 | 22% | 7% | 9% | 18% | 6% |
| 9 | Blekinge | 1.1 | 1.0 | 17% | 7% | 14% | 20% | 6% |
| 10 | Blekinge | 1.1 | 1.0 | 18% | 6% | 10% | 19% | 9% |
| 11 | Blekinge | 1.1 | 1.0 | 19% | 5% | 7% | 19% | 11% |
| 12 | Blekinge | 0.9 | 0.8 | 14% | 6% | 7% | 18% | 8% |
| 13 | Blekinge | 0.8 | 0.7 | 9% | 4% | 12% | 15% | 6% |
| 14 | Blekinge | 0.8 | 0.7 | 11% | 5% | 11% | 12% | 5% |
| 15 | Blekinge | 0.7 | 0.6 | 4% | 5% | 10% | 14% | 6% |
| 16 | Blekinge | 0.6 | 0.5 | 3% | 5% | 11% | 10% | 7% |
| 17 | Blekinge | 0.5 | 0.5 | 2% | 5% | 9% | 11% | 4% |
| 18 | Blekinge | 0.4 | 0.3 | 2% | 4% | 9% | 4% | 4% |
| 19 | Dalarna | 3.2 | 3.6 | 70% | 17% | 6% | 45% | 37% |
| 20 | Dalarna | 2.0 | 2.2 | 35% | 13% | 13% | 31% | 18% |
| 21 | Dalarna | 1.7 | 1.8 | 31% | 11% | 7% | 25% | 17% |
| 22 | Dalarna | 1.6 | 1.8 | 32% | 10% | 6% | 25% | 17% |
| 23 | Dalarna | 1.5 | 1.7 | 34% | 8% | 5% | 23% | 13% |
| 24 | Dalarna | 1.5 | 1.6 | 27% | 9% | 10% | 22% | 13% |
| 25 | Dalarna | 1.3 | 1.4 | 20% | 9% | 4% | 21% | 19% |
| 26 | Dalarna | 1.3 | 1.4 | 21% | 8% | 4% | 20% | 18% |
| 27 | Dalarna | 1.2 | 1.4 | 21% | 9% | 7% | 20% | 12% |
| 28 | Dalarna | 1.1 | 1.2 | 18% | 8% | 14% | 15% | 9% |
| 29 | Dalarna | 1.1 | 1.2 | 11% | 10% | 11% | 20% | 11% |
| 30 | Dalarna | 1.1 | 1.2 | 16% | 6% | 12% | 16% | 11% |
| 31 | Dalarna | 1.0 | 1.1 | 11% | 7% | 12% | 15% | 11% |
| 32 | Dalarna | 0.9 | 1.0 | 14% | 7% | 11% | 13% | 7% |
| 33 | Dalarna | 0.9 | 1.0 | 2% | 5% | 16% | 18% | 12% |
| 34 | Dalarna | 0.9 | 1.0 | 12% | 6% | 5% | 12% | 15% |
| 35 | Dalarna | 0.9 | 1.0 | 14% | 8% | 5% | 13% | 8% |
| 36 | Dalarna | 0.8 | 0.9 | 8% | 6% | 11% | 13% | 8% |
| 37 | Dalarna | 0.8 | 0.9 | 3% | 13% | 8% | 17% | 6% |
| 38 | Dalarna | 0.8 | 0.8 | 7% | 6% | 8% | 13% | 10% |
| 39 | Dalarna | 0.8 | 0.8 | 8% | 8% | 5% | 16% | 7% |
| 40 | Dalarna | 0.7 | 0.8 | 9% | 5% | 10% | 12% | 6% |
| 41 | Dalarna | 0.7 | 0.8 | 7% | 6% | 4% | 12% | 11% |
| 42 | Dalarna | 0.7 | 0.8 | 8% | 7% | 6% | 11% | 8% |
| 43 | Dalarna | 0.7 | 0.7 | 7% | 8% | 6% | 10% | 8% |
| 44 | Dalarna | 0.6 | 0.6 | 4% | 4% | 9% | 10% | 5% |
| 45 | Dalarna | 0.5 | 0.6 | 4% | 5% | 13% | 6% | 3% |
| 46 | Dalarna | 0.5 | 0.5 | 3% | 4% | 8% | 7% | 5% |
| 47 | Gotland | 0.9 | 1.2 | 9% | 8% | 12% | 17% | 8% |
| 48 | Gotland | 0.8 | 1.0 | 7% | 6% | 14% | 12% | 6% |
| 49 | Gotland | 0.7 | 0.9 | 6% | 6% | 11% | 13% | 7% |
| 50 | Gävleborg | 2.6 | 2.0 | 53% | 15% | 12% | 41% | 23% |
| 51 | Gävleborg | 2.5 | 1.9 | 50% | 12% | 15% | 39% | 20% |
| 52 | Gävleborg | 2.2 | 1.7 | 41% | 11% | 13% | 37% | 18% |
| 53 | Gävleborg | 2.0 | 1.5 | 36% | 8% | 11% | 34% | 18% |
| 54 | Gävleborg | 1.8 | 1.4 | 30% | 9% | 12% | 34% | 18% |
| 55 | Gävleborg | 1.8 | 1.4 | 32% | 10% | 10% | 31% | 15% |
| 56 | Gävleborg | 1.7 | 1.3 | 28% | 10% | 3% | 32% | 23% |
| 57 | Gävleborg | 1.7 | 1.3 | 31% | 8% | 8% | 30% | 17% |
| 58 | Gävleborg | 1.7 | 1.3 | 28% | 8% | 18% | 26% | 15% |
| 59 | Gävleborg | 1.5 | 1.2 | 27% | 9% | 6% | 29% | 14% |
| 60 | Gävleborg | 1.5 | 1.2 | 25% | 8% | 16% | 23% | 12% |
| 61 | Gävleborg | 1.5 | 1.2 | 20% | 9% | 25% | 21% | 9% |
| 62 | Gävleborg | 1.4 | 1.1 | 21% | 8% | 8% | 26% | 13% |
| 63 | Gävleborg | 1.3 | 1.0 | 19% | 8% | 8% | 24% | 13% |
| 64 | Gävleborg | 1.3 | 1.0 | 17% | 11% | 10% | 22% | 13% |
| 65 | Gävleborg | 1.2 | 0.9 | 13% | 4% | 11% | 26% | 12% |
| 66 | Gävleborg | 1.2 | 0.9 | 19% | 6% | 6% | 21% | 12% |
| 67 | Gävleborg | 1.1 | 0.9 | 14% | 9% | 9% | 22% | 11% |
| 68 | Gävleborg | 1.1 | 0.9 | 9% | 11% | 10% | 24% | 10% |
| 69 | Gävleborg | 1.0 | 0.8 | 13% | 7% | 13% | 16% | 8% |
| 70 | Gävleborg | 1.0 | 0.8 | 14% | 6% | 11% | 17% | 7% |
| 71 | Gävleborg | 0.9 | 0.7 | 8% | 12% | 6% | 19% | 9% |
| 72 | Gävleborg | 0.9 | 0.7 | 9% | 7% | 4% | 19% | 11% |
| 73 | Gävleborg | 0.9 | 0.7 | 5% | 9% | 10% | 18% | 9% |
| 74 | Gävleborg | 0.9 | 0.7 | 7% | 10% | 9% | 17% | 8% |
| 75 | Gävleborg | 0.9 | 0.7 | 12% | 7% | 13% | 12% | 6% |
| 76 | Gävleborg | 0.8 | 0.7 | 11% | 5% | 15% | 12% | 5% |
| 77 | Gävleborg | 0.8 | 0.6 | 5% | 8% | 8% | 18% | 9% |
| 78 | Gävleborg | 0.8 | 0.6 | 6% | 5% | 11% | 16% | 7% |
| 79 | Gävleborg | 0.7 | 0.6 | 5% | 4% | 8% | 16% | 11% |
| 80 | Gävleborg | 0.7 | 0.5 | 3% | 7% | 4% | 17% | 7% |
| 81 | Gävleborg | 0.6 | 0.5 | 6% | 5% | 10% | 12% | 6% |
| 82 | Gävleborg | 0.5 | 0.4 | 4% | 4% | 10% | 8% | 4% |
| 83 | Halland | 2.9 | 3.7 | 63% | 7% | 11% | 50% | 23% |
| 84 | Halland | 2.1 | 2.8 | 43% | 9% | 11% | 37% | 15% |
| 85 | Halland | 1.9 | 2.5 | 35% | 9% | 13% | 34% | 14% |
| 86 | Halland | 1.5 | 2.0 | 28% | 6% | 12% | 26% | 13% |
| 87 | Halland | 1.5 | 1.9 | 26% | 6% | 10% | 24% | 15% |
| 88 | Halland | 1.4 | 1.8 | 24% | 5% | 16% | 23% | 11% |
| 89 | Halland | 1.3 | 1.7 | 21% | 5% | 13% | 22% | 11% |
| 90 | Halland | 1.3 | 1.6 | 22% | 7% | 11% | 21% | 10% |
| 91 | Halland | 1.2 | 1.6 | 18% | 7% | 12% | 21% | 12% |
| 92 | Halland | 1.1 | 1.5 | 15% | 6% | 14% | 19% | 11% |
| 93 | Halland | 1.1 | 1.5 | 15% | 7% | 15% | 19% | 6% |
| 94 | Halland | 1.1 | 1.5 | 15% | 5% | 12% | 19% | 12% |
| 95 | Halland | 1.0 | 1.4 | 7% | 7% | 18% | 16% | 13% |
| 96 | Halland | 0.9 | 1.2 | 12% | 5% | 14% | 15% | 6% |
| 97 | Halland | 0.9 | 1.2 | 10% | 5% | 16% | 16% | 5% |
| 98 | Halland | 0.9 | 1.1 | 12% | 5% | 17% | 10% | 7% |
| 99 | Halland | 0.8 | 1.1 | 7% | 3% | 21% | 15% | 3% |
| 100 | Halland | 0.8 | 1.1 | 9% | 6% | 15% | 9% | 10% |
| 101 | Halland | 0.8 | 1.1 | 10% | 5% | 14% | 12% | 6% |
| 102 | Halland | 0.8 | 1.0 | 7% | 7% | 10% | 16% | 7% |
| 103 | Halland | 0.8 | 1.0 | 8% | 4% | 8% | 15% | 9% |
| 104 | Halland | 0.8 | 1.0 | 9% | 5% | 12% | 11% | 9% |
| 105 | Halland | 0.8 | 1.0 | 9% | 4% | 18% | 10% | 5% |
| 106 | Halland | 0.8 | 1.0 | 9% | 4% | 13% | 11% | 6% |
| 107 | Halland | 0.7 | 1.0 | 9% | 5% | 14% | 11% | 4% |
| 108 | Halland | 0.7 | 1.0 | 9% | 4% | 14% | 10% | 5% |
| 109 | Halland | 0.7 | 0.9 | 8% | 4% | 12% | 9% | 6% |
| 110 | Halland | 0.7 | 0.9 | 8% | 3% | 16% | 8% | 2% |
| 111 | Halland | 0.6 | 0.8 | 7% | 4% | 14% | 8% | 5% |
| 112 | Halland | 0.6 | 0.8 | 9% | 6% | 7% | 9% | 3% |
| 113 | Halland | 0.6 | 0.8 | 4% | 3% | 16% | 9% | 4% |
| 114 | Halland | 0.6 | 0.8 | 6% | 4% | 8% | 10% | 7% |
| 115 | Halland | 0.6 | 0.7 | 2% | 5% | 18% | 7% | 3% |
| 116 | Halland | 0.6 | 0.7 | 5% | 3% | 11% | 8% | 6% |
| 117 | Halland | 0.5 | 0.7 | 4% | 4% | 14% | 8% | 3% |
| 118 | Halland | 0.5 | 0.7 | 6% | 5% | 11% | 8% | 2% |
| 119 | Halland | 0.5 | 0.7 | 4% | 4% | 12% | 8% | 4% |
| 120 | Halland | 0.5 | 0.7 | 5% | 3% | 11% | 7% | 5% |
| 121 | Halland | 0.5 | 0.7 | 4% | 3% | 13% | 7% | 4% |
| 122 | Halland | 0.5 | 0.6 | 3% | 3% | 12% | 8% | 4% |
| 123 | Halland | 0.5 | 0.6 | 2% | 3% | 16% | 4% | 3% |
| 124 | Halland | 0.5 | 0.6 | 3% | 3% | 12% | 5% | 5% |
| 125 | Halland | 0.4 | 0.5 | 4% | 3% | 10% | 6% | 2% |
| 126 | Halland | 0.4 | 0.5 | 3% | 3% | 10% | 6% | 2% |
| 127 | Halland | 0.4 | 0.5 | 2% | 4% | 10% | 6% | 4% |
| 128 | Halland | 0.4 | 0.5 | 3% | 2% | 15% | 4% | 2% |
| 129 | Halland | 0.4 | 0.5 | 2% | 3% | 13% | 4% | 1% |
| 130 | Jämtland | 1.6 | 2.0 | 29% | 6% | 14% | 23% | 18% |
| 131 | Jämtland | 1.3 | 1.7 | 21% | 6% | 9% | 25% | 12% |
| 132 | Jämtland | 1.3 | 1.6 | 18% | 13% | 9% | 21% | 15% |
| 133 | Jämtland | 1.3 | 1.6 | 21% | 7% | 8% | 23% | 12% |
| 134 | Jämtland | 1.3 | 1.6 | 22% | 8% | 10% | 21% | 9% |
| 135 | Jämtland | 1.3 | 1.6 | 19% | 10% | 8% | 20% | 14% |
| 136 | Jämtland | 1.1 | 1.4 | 19% | 6% | 6% | 18% | 15% |
| 137 | Jämtland | 1.1 | 1.4 | 17% | 7% | 14% | 18% | 8% |
| 138 | Jämtland | 1.1 | 1.4 | 12% | 10% | 10% | 16% | 15% |
| 139 | Jämtland | 1.1 | 1.3 | 10% | 9% | 10% | 23% | 9% |
| 140 | Jämtland | 1.0 | 1.3 | 11% | 7% | 19% | 18% | 6% |
| 141 | Jämtland | 1.0 | 1.3 | 13% | 5% | 16% | 18% | 5% |
| 142 | Jämtland | 0.9 | 1.1 | 11% | 6% | 12% | 13% | 8% |
| 143 | Jämtland | 0.8 | 1.0 | 12% | 5% | 8% | 13% | 8% |
| 144 | Jämtland | 0.8 | 1.0 | 8% | 8% | 11% | 13% | 7% |
| 145 | Jämtland | 0.8 | 1.0 | 10% | 6% | 15% | 11% | 4% |
| 146 | Jämtland | 0.8 | 1.0 | 9% | 6% | 12% | 13% | 4% |
| 147 | Jämtland | 0.7 | 0.9 | 6% | 4% | 3% | 17% | 10% |
| 148 | Jämtland | 0.6 | 0.8 | 3% | 7% | 12% | 11% | 6% |
| 149 | Jämtland | 0.6 | 0.8 | 2% | 10% | 10% | 9% | 5% |
| 150 | Jämtland | 0.6 | 0.8 | 4% | 10% | 7% | 8% | 8% |
| 151 | Jämtland | 0.6 | 0.7 | 4% | 5% | 12% | 11% | 3% |
| 152 | Jämtland | 0.6 | 0.7 | 6% | 5% | 12% | 7% | 5% |
| 153 | Jämtland | 0.6 | 0.7 | 4% | 4% | 13% | 10% | 2% |
| 154 | Jämtland | 0.5 | 0.7 | 2% | 4% | 7% | 12% | 6% |
| 155 | Jämtland | 0.5 | 0.6 | 1% | 3% | 11% | 8% | 4% |
| 156 | Jönköping | 2.7 | 2.6 | 66% | 9% | 14% | 34% | 22% |
| 157 | Jönköping | 1.7 | 1.7 | 40% | 5% | 14% | 21% | 14% |
| 158 | Jönköping | 1.5 | 1.5 | 29% | 7% | 10% | 24% | 14% |
| 159 | Jönköping | 1.5 | 1.5 | 27% | 7% | 8% | 25% | 18% |
| 160 | Jönköping | 1.5 | 1.4 | 22% | 8% | 11% | 29% | 13% |
| 161 | Jönköping | 1.3 | 1.2 | 26% | 6% | 13% | 16% | 10% |
| 162 | Jönköping | 1.2 | 1.2 | 23% | 4% | 10% | 19% | 12% |
| 163 | Jönköping | 1.2 | 1.1 | 21% | 5% | 9% | 18% | 13% |
| 164 | Jönköping | 1.2 | 1.1 | 21% | 3% | 12% | 19% | 9% |
| 165 | Jönköping | 1.1 | 1.1 | 20% | 7% | 6% | 19% | 10% |
| 166 | Jönköping | 1.1 | 1.0 | 20% | 6% | 7% | 14% | 13% |
| 167 | Jönköping | 1.1 | 1.0 | 18% | 5% | 8% | 17% | 14% |
| 168 | Jönköping | 1.0 | 1.0 | 19% | 5% | 8% | 16% | 9% |
| 169 | Jönköping | 1.0 | 1.0 | 19% | 4% | 13% | 13% | 8% |
| 170 | Jönköping | 1.0 | 0.9 | 16% | 5% | 8% | 15% | 10% |
| 171 | Jönköping | 0.9 | 0.9 | 15% | 5% | 9% | 15% | 7% |
| 172 | Jönköping | 0.8 | 0.8 | 12% | 4% | 12% | 12% | 7% |
| 173 | Jönköping | 0.8 | 0.8 | 14% | 5% | 10% | 10% | 5% |
| 174 | Jönköping | 0.8 | 0.8 | 13% | 4% | 15% | 9% | 5% |
| 175 | Jönköping | 0.8 | 0.8 | 11% | 4% | 14% | 9% | 8% |
| 176 | Jönköping | 0.7 | 0.7 | 12% | 3% | 12% | 10% | 4% |
| 177 | Jönköping | 0.6 | 0.6 | 10% | 3% | 15% | 7% | 4% |
| 178 | Jönköping | 0.6 | 0.5 | 4% | 3% | 12% | 9% | 4% |
| 179 | Jönköping | 0.5 | 0.5 | 6% | 2% | 13% | 6% | 4% |
| 180 | Jönköping | 0.5 | 0.5 | 3% | 4% | 11% | 6% | 5% |
| 181 | Kalmar | 2.1 | 2.2 | 45% | 6% | 17% | 31% | 17% |
| 182 | Kalmar | 2.1 | 2.2 | 35% | 8% | 22% | 32% | 20% |
| 183 | Kalmar | 2.0 | 2.1 | 36% | 6% | 14% | 34% | 23% |
| 184 | Kalmar | 1.9 | 1.9 | 33% | 7% | 10% | 33% | 21% |
| 185 | Kalmar | 1.6 | 1.6 | 30% | 6% | 15% | 25% | 11% |
| 186 | Kalmar | 1.4 | 1.4 | 24% | 6% | 7% | 24% | 17% |
| 187 | Kalmar | 1.4 | 1.4 | 25% | 5% | 15% | 20% | 9% |
| 188 | Kalmar | 1.3 | 1.3 | 25% | 7% | 7% | 21% | 13% |
| 189 | Kalmar | 1.3 | 1.3 | 17% | 3% | 8% | 27% | 17% |
| 190 | Kalmar | 1.3 | 1.3 | 22% | 6% | 9% | 21% | 14% |
| 191 | Kalmar | 1.3 | 1.3 | 22% | 6% | 10% | 21% | 12% |
| 192 | Kalmar | 1.3 | 1.3 | 23% | 5% | 7% | 21% | 14% |
| 193 | Kalmar | 1.3 | 1.3 | 22% | 6% | 9% | 21% | 13% |
| 194 | Kalmar | 1.1 | 1.2 | 14% | 7% | 9% | 21% | 13% |
| 195 | Kalmar | 1.1 | 1.1 | 17% | 4% | 14% | 17% | 8% |
| 196 | Kalmar | 1.1 | 1.1 | 21% | 8% | 7% | 14% | 8% |
| 197 | Kalmar | 1.0 | 1.0 | 17% | 5% | 7% | 15% | 11% |
| 198 | Kalmar | 1.0 | 1.0 | 13% | 7% | 17% | 12% | 6% |
| 199 | Kalmar | 1.0 | 1.0 | 14% | 6% | 8% | 15% | 12% |
| 200 | Kalmar | 0.9 | 1.0 | 12% | 5% | 13% | 17% | 6% |
| 201 | Kalmar | 0.9 | 1.0 | 10% | 8% | 11% | 14% | 10% |
| 202 | Kalmar | 0.9 | 0.9 | 16% | 5% | 5% | 15% | 10% |
| 203 | Kalmar | 0.9 | 0.9 | 13% | 3% | 9% | 16% | 11% |
| 204 | Kalmar | 0.9 | 0.9 | 13% | 5% | 6% | 15% | 10% |
| 205 | Kalmar | 0.9 | 0.9 | 11% | 5% | 9% | 15% | 11% |
| 206 | Kalmar | 0.9 | 0.9 | 10% | 8% | 9% | 14% | 8% |
| 207 | Kalmar | 0.8 | 0.9 | 7% | 4% | 10% | 15% | 14% |
| 208 | Kalmar | 0.8 | 0.9 | 9% | 6% | 18% | 11% | 5% |
| 209 | Kalmar | 0.8 | 0.8 | 7% | 6% | 9% | 15% | 11% |
| 210 | Kalmar | 0.7 | 0.8 | 10% | 4% | 14% | 10% | 5% |
| 211 | Kalmar | 0.7 | 0.7 | 9% | 3% | 17% | 7% | 3% |
| 212 | Kalmar | 0.7 | 0.7 | 10% | 3% | 10% | 10% | 4% |
| 213 | Kalmar | 0.6 | 0.7 | 7% | 5% | 9% | 10% | 5% |
| 214 | Kalmar | 0.6 | 0.6 | 5% | 3% | 13% | 9% | 4% |
| 215 | Kalmar | 0.5 | 0.5 | 5% | 3% | 11% | 7% | 4% |
| 216 | Kalmar | 0.5 | 0.5 | 4% | 4% | 6% | 8% | 5% |
| 217 | Kalmar | 0.4 | 0.4 | 2% | 3% | 8% | 5% | 4% |
| 218 | Kronoberg | 3.1 | 3.0 | 67% | 10% | 18% | 50% | 24% |
| 219 | Kronoberg | 2.1 | 2.1 | 43% | 4% | 10% | 38% | 23% |
| 220 | Kronoberg | 1.9 | 1.9 | 35% | 6% | 10% | 34% | 20% |
| 221 | Kronoberg | 1.9 | 1.8 | 32% | 6% | 13% | 29% | 26% |
| 222 | Kronoberg | 1.9 | 1.8 | 32% | 9% | 12% | 32% | 18% |
| 223 | Kronoberg | 1.6 | 1.6 | 28% | 6% | 10% | 33% | 13% |
| 224 | Kronoberg | 1.6 | 1.5 | 28% | 7% | 8% | 25% | 20% |
| 225 | Kronoberg | 1.5 | 1.4 | 28% | 6% | 5% | 26% | 16% |
| 226 | Kronoberg | 1.5 | 1.4 | 26% | 7% | 8% | 24% | 15% |
| 227 | Kronoberg | 1.4 | 1.4 | 31% | 5% | 13% | 21% | 8% |
| 228 | Kronoberg | 1.4 | 1.3 | 23% | 7% | 22% | 18% | 7% |
| 229 | Kronoberg | 1.4 | 1.3 | 25% | 7% | 8% | 23% | 12% |
| 230 | Kronoberg | 1.3 | 1.3 | 20% | 10% | 10% | 22% | 14% |
| 231 | Kronoberg | 1.3 | 1.3 | 29% | 4% | 8% | 20% | 9% |
| 232 | Kronoberg | 1.1 | 1.1 | 18% | 7% | 10% | 16% | 12% |
| 233 | Kronoberg | 1.1 | 1.0 | 15% | 5% | 8% | 18% | 14% |
| 234 | Kronoberg | 1.0 | 1.0 | 21% | 3% | 9% | 16% | 6% |
| 235 | Kronoberg | 1.0 | 1.0 | 17% | 4% | 8% | 18% | 9% |
| 236 | Kronoberg | 1.0 | 0.9 | 15% | 4% | 8% | 18% | 9% |
| 237 | Kronoberg | 1.0 | 0.9 | 12% | 7% | 18% | 13% | 5% |
| 238 | Kronoberg | 0.9 | 0.8 | 14% | 4% | 4% | 15% | 11% |
| 239 | Kronoberg | 0.8 | 0.8 | 11% | 5% | 9% | 14% | 7% |
| 240 | Kronoberg | 0.8 | 0.8 | 8% | 6% | 9% | 13% | 10% |
| 241 | Kronoberg | 0.8 | 0.7 | 10% | 4% | 10% | 13% | 7% |
| 242 | Kronoberg | 0.8 | 0.7 | 9% | 4% | 12% | 13% | 7% |
| 243 | Kronoberg | 0.7 | 0.7 | 10% | 4% | 12% | 12% | 5% |
| 244 | Kronoberg | 0.7 | 0.7 | 11% | 3% | 13% | 9% | 5% |
| 245 | Kronoberg | 0.7 | 0.7 | 10% | 4% | 9% | 12% | 4% |
| 246 | Kronoberg | 0.7 | 0.7 | 8% | 4% | 14% | 9% | 4% |
| 247 | Kronoberg | 0.7 | 0.6 | 7% | 5% | 10% | 11% | 5% |
| 248 | Kronoberg | 0.6 | 0.6 | 7% | 4% | 15% | 7% | 4% |
| 249 | Kronoberg | 0.6 | 0.5 | 6% | 4% | 9% | 8% | 5% |
| 250 | Norrbotten | 1.6 | 2.0 | 30% | 8% | 12% | 26% | 13% |
| 251 | Norrbotten | 1.2 | 1.4 | 14% | 9% | 6% | 24% | 14% |
| 252 | Norrbotten | 1.1 | 1.4 | 19% | 9% | 6% | 19% | 10% |
| 253 | Norrbotten | 1.1 | 1.3 | 18% | 7% | 10% | 18% | 7% |
| 254 | Norrbotten | 1.0 | 1.2 | 16% | 8% | 12% | 11% | 9% |
| 255 | Norrbotten | 0.9 | 1.1 | 10% | 7% | 7% | 19% | 9% |
| 256 | Norrbotten | 0.9 | 1.1 | 18% | 5% | 12% | 12% | 4% |
| 257 | Norrbotten | 0.9 | 1.1 | 13% | 6% | 11% | 15% | 7% |
| 258 | Norrbotten | 0.9 | 1.1 | 12% | 6% | 13% | 13% | 7% |
| 259 | Norrbotten | 0.9 | 1.1 | 8% | 7% | 7% | 16% | 13% |
| 260 | Norrbotten | 0.9 | 1.1 | 8% | 7% | 5% | 20% | 10% |
| 261 | Norrbotten | 0.8 | 1.0 | 10% | 6% | 6% | 17% | 8% |
| 262 | Norrbotten | 0.8 | 1.0 | 10% | 8% | 5% | 16% | 10% |
| 263 | Norrbotten | 0.8 | 1.0 | 7% | 7% | 20% | 11% | 5% |
| 264 | Norrbotten | 0.8 | 1.0 | 13% | 4% | 10% | 10% | 7% |
| 265 | Norrbotten | 0.8 | 0.9 | 7% | 7% | 7% | 10% | 14% |
| 266 | Norrbotten | 0.8 | 0.9 | 8% | 5% | 5% | 14% | 12% |
| 267 | Norrbotten | 0.8 | 0.9 | 8% | 7% | 4% | 15% | 9% |
| 268 | Norrbotten | 0.7 | 0.9 | 8% | 6% | 7% | 16% | 6% |
| 269 | Norrbotten | 0.7 | 0.8 | 8% | 9% | 9% | 8% | 6% |
| 270 | Norrbotten | 0.7 | 0.8 | 5% | 6% | 11% | 11% | 6% |
| 271 | Norrbotten | 0.7 | 0.8 | 7% | 6% | 8% | 13% | 5% |
| 272 | Norrbotten | 0.7 | 0.8 | 11% | 7% | 3% | 8% | 9% |
| 273 | Norrbotten | 0.7 | 0.8 | 7% | 5% | 12% | 10% | 4% |
| 274 | Norrbotten | 0.7 | 0.8 | 7% | 5% | 6% | 12% | 8% |
| 275 | Norrbotten | 0.6 | 0.8 | 7% | 4% | 9% | 10% | 5% |
| 276 | Norrbotten | 0.6 | 0.7 | 5% | 4% | 9% | 9% | 5% |
| 277 | Norrbotten | 0.4 | 0.5 | 2% | 3% | 6% | 9% | 4% |
| 278 | Skåne | 3.6 | 3.2 | 76% | 12% | 13% | 62% | 31% |
| 279 | Skåne | 3.3 | 3.0 | 64% | 9% | 28% | 55% | 25% |
| 280 | Skåne | 3.3 | 3.0 | 68% | 14% | 14% | 54% | 29% |
| 281 | Skåne | 3.0 | 2.7 | 65% | 11% | 13% | 48% | 23% |
| 282 | Skåne | 2.9 | 2.6 | 63% | 10% | 13% | 50% | 23% |
| 283 | Skåne | 2.9 | 2.6 | 61% | 7% | 18% | 49% | 23% |
| 284 | Skåne | 2.9 | 2.6 | 61% | 11% | 13% | 48% | 25% |
| 285 | Skåne | 2.8 | 2.6 | 61% | 9% | 16% | 48% | 18% |
| 286 | Skåne | 2.7 | 2.5 | 59% | 8% | 16% | 43% | 20% |
| 287 | Skåne | 2.6 | 2.3 | 39% | 20% | 28% | 41% | 19% |
| 288 | Skåne | 2.5 | 2.2 | 44% | 13% | 19% | 43% | 18% |
| 289 | Skåne | 2.5 | 2.2 | 42% | 9% | 15% | 43% | 28% |
| 290 | Skåne | 2.3 | 2.1 | 44% | 9% | 18% | 40% | 17% |
| 291 | Skåne | 2.3 | 2.1 | 42% | 11% | 16% | 40% | 21% |
| 292 | Skåne | 2.2 | 2.0 | 46% | 12% | 18% | 29% | 17% |
| 293 | Skåne | 2.2 | 2.0 | 34% | 10% | 12% | 40% | 26% |
| 294 | Skåne | 2.2 | 1.9 | 41% | 8% | 20% | 37% | 12% |
| 295 | Skåne | 2.1 | 1.9 | 47% | 6% | 13% | 32% | 16% |
| 296 | Skåne | 2.1 | 1.9 | 36% | 10% | 18% | 33% | 18% |
| 297 | Skåne | 2.1 | 1.9 | 41% | 10% | 15% | 33% | 13% |
| 298 | Skåne | 2.0 | 1.8 | 38% | 9% | 10% | 36% | 19% |
| 299 | Skåne | 1.9 | 1.7 | 34% | 9% | 7% | 36% | 19% |
| 300 | Skåne | 1.9 | 1.7 | 37% | 8% | 15% | 31% | 15% |
| 301 | Skåne | 1.9 | 1.7 | 38% | 8% | 11% | 33% | 15% |
| 302 | Skåne | 1.9 | 1.7 | 37% | 10% | 12% | 31% | 13% |
| 303 | Skåne | 1.8 | 1.7 | 27% | 8% | 12% | 35% | 21% |
| 304 | Skåne | 1.8 | 1.6 | 38% | 5% | 14% | 29% | 12% |
| 305 | Skåne | 1.8 | 1.6 | 33% | 7% | 22% | 28% | 9% |
| 306 | Skåne | 1.8 | 1.6 | 32% | 9% | 18% | 29% | 9% |
| 307 | Skåne | 1.7 | 1.6 | 31% | 8% | 10% | 31% | 16% |
| 308 | Skåne | 1.7 | 1.6 | 32% | 8% | 12% | 29% | 15% |
| 309 | Skåne | 1.7 | 1.5 | 30% | 7% | 19% | 29% | 10% |
| 310 | Skåne | 1.7 | 1.5 | 26% | 10% | 22% | 27% | 10% |
| 311 | Skåne | 1.6 | 1.5 | 22% | 10% | 15% | 29% | 16% |
| 312 | Skåne | 1.6 | 1.5 | 28% | 10% | 17% | 25% | 10% |
| 313 | Skåne | 1.6 | 1.5 | 27% | 10% | 18% | 26% | 10% |
| 314 | Skåne | 1.6 | 1.4 | 34% | 7% | 14% | 23% | 8% |
| 315 | Skåne | 1.6 | 1.4 | 29% | 6% | 23% | 23% | 7% |
| 316 | Skåne | 1.6 | 1.4 | 29% | 8% | 15% | 25% | 10% |
| 317 | Skåne | 1.6 | 1.4 | 36% | 7% | 16% | 21% | 6% |
| 318 | Skåne | 1.5 | 1.4 | 22% | 7% | 13% | 28% | 16% |
| 319 | Skåne | 1.5 | 1.3 | 23% | 5% | 19% | 25% | 11% |
| 320 | Skåne | 1.5 | 1.3 | 26% | 8% | 13% | 24% | 11% |
| 321 | Skåne | 1.5 | 1.3 | 26% | 8% | 16% | 24% | 7% |
| 322 | Skåne | 1.5 | 1.3 | 28% | 7% | 13% | 22% | 11% |
| 323 | Skåne | 1.4 | 1.3 | 32% | 7% | 13% | 21% | 4% |
| 324 | Skåne | 1.4 | 1.2 | 21% | 6% | 9% | 27% | 14% |
| 325 | Skåne | 1.3 | 1.2 | 18% | 7% | 12% | 25% | 13% |
| 326 | Skåne | 1.3 | 1.2 | 25% | 4% | 8% | 25% | 12% |
| 327 | Skåne | 1.3 | 1.2 | 20% | 6% | 18% | 19% | 11% |
| 328 | Skåne | 1.3 | 1.2 | 26% | 7% | 16% | 17% | 5% |
| 329 | Skåne | 1.3 | 1.2 | 18% | 8% | 15% | 20% | 13% |
| 330 | Skåne | 1.3 | 1.2 | 14% | 9% | 12% | 26% | 14% |
| 331 | Skåne | 1.3 | 1.2 | 16% | 7% | 17% | 23% | 10% |
| 332 | Skåne | 1.3 | 1.1 | 17% | 7% | 10% | 25% | 13% |
| 333 | Skåne | 1.3 | 1.1 | 18% | 7% | 9% | 22% | 13% |
| 334 | Skåne | 1.2 | 1.1 | 19% | 7% | 8% | 21% | 14% |
| 335 | Skåne | 1.2 | 1.1 | 23% | 6% | 17% | 17% | 5% |
| 336 | Skåne | 1.2 | 1.1 | 19% | 6% | 19% | 19% | 6% |
| 337 | Skåne | 1.2 | 1.1 | 16% | 8% | 9% | 22% | 15% |
| 338 | Skåne | 1.2 | 1.1 | 7% | 15% | 16% | 21% | 14% |
| 339 | Skåne | 1.2 | 1.1 | 11% | 9% | 18% | 17% | 15% |
| 340 | Skåne | 1.2 | 1.1 | 15% | 6% | 9% | 22% | 16% |
| 341 | Skåne | 1.2 | 1.1 | 17% | 9% | 7% | 23% | 11% |
| 342 | Skåne | 1.2 | 1.0 | 15% | 7% | 11% | 20% | 13% |
| 343 | Skåne | 1.2 | 1.0 | 21% | 5% | 15% | 16% | 7% |
| 344 | Skåne | 1.1 | 1.0 | 14% | 6% | 17% | 19% | 9% |
| 345 | Skåne | 1.1 | 1.0 | 13% | 4% | 20% | 19% | 9% |
| 346 | Skåne | 1.1 | 1.0 | 19% | 7% | 13% | 18% | 6% |
| 347 | Skåne | 1.1 | 1.0 | 16% | 7% | 18% | 18% | 5% |
| 348 | Skåne | 1.1 | 1.0 | 16% | 5% | 9% | 20% | 14% |
| 349 | Skåne | 1.1 | 1.0 | 17% | 7% | 9% | 21% | 8% |
| 350 | Skåne | 1.1 | 1.0 | 16% | 7% | 19% | 16% | 5% |
| 351 | Skåne | 1.1 | 1.0 | 9% | 9% | 13% | 18% | 16% |
| 352 | Skåne | 1.1 | 1.0 | 15% | 6% | 16% | 17% | 9% |
| 353 | Skåne | 1.1 | 0.9 | 15% | 6% | 16% | 16% | 7% |
| 354 | Skåne | 1.0 | 0.9 | 12% | 5% | 12% | 19% | 11% |
| 355 | Skåne | 1.0 | 0.9 | 6% | 9% | 14% | 21% | 9% |
| 356 | Skåne | 1.0 | 0.9 | 17% | 5% | 17% | 14% | 3% |
| 357 | Skåne | 1.0 | 0.9 | 21% | 5% | 13% | 13% | 4% |
| 358 | Skåne | 1.0 | 0.9 | 12% | 6% | 15% | 16% | 8% |
| 359 | Skåne | 1.0 | 0.9 | 3% | 10% | 22% | 18% | 8% |
| 360 | Skåne | 1.0 | 0.9 | 16% | 5% | 15% | 14% | 5% |
| 361 | Skåne | 1.0 | 0.9 | 13% | 5% | 12% | 17% | 7% |
| 362 | Skåne | 1.0 | 0.9 | 17% | 5% | 18% | 11% | 3% |
| 363 | Skåne | 1.0 | 0.9 | 12% | 7% | 16% | 15% | 5% |
| 364 | Skåne | 1.0 | 0.9 | 14% | 5% | 14% | 16% | 5% |
| 365 | Skåne | 1.0 | 0.9 | 10% | 3% | 13% | 16% | 14% |
| 366 | Skåne | 1.0 | 0.9 | 8% | 7% | 10% | 20% | 11% |
| 367 | Skåne | 0.9 | 0.8 | 9% | 7% | 12% | 17% | 11% |
| 368 | Skåne | 0.9 | 0.8 | 15% | 5% | 14% | 13% | 6% |
| 369 | Skåne | 0.9 | 0.8 | 11% | 7% | 17% | 14% | 3% |
| 370 | Skåne | 0.9 | 0.8 | 11% | 5% | 12% | 16% | 8% |
| 371 | Skåne | 0.9 | 0.8 | 11% | 7% | 9% | 16% | 8% |
| 372 | Skåne | 0.9 | 0.8 | 8% | 6% | 9% | 19% | 9% |
| 373 | Skåne | 0.9 | 0.8 | 13% | 4% | 13% | 13% | 8% |
| 374 | Skåne | 0.9 | 0.8 | 11% | 5% | 16% | 14% | 6% |
| 375 | Skåne | 0.9 | 0.8 | 15% | 6% | 16% | 11% | 3% |
| 376 | Skåne | 0.9 | 0.8 | 9% | 6% | 17% | 14% | 6% |
| 377 | Skåne | 0.9 | 0.8 | 12% | 6% | 15% | 14% | 3% |
| 378 | Skåne | 0.9 | 0.8 | 10% | 5% | 13% | 15% | 8% |
| 379 | Skåne | 0.9 | 0.8 | 11% | 5% | 13% | 16% | 6% |
| 380 | Skåne | 0.9 | 0.8 | 12% | 6% | 8% | 16% | 9% |
| 381 | Skåne | 0.9 | 0.8 | 11% | 5% | 11% | 16% | 6% |
| 382 | Skåne | 0.9 | 0.8 | 8% | 5% | 13% | 15% | 9% |
| 383 | Skåne | 0.9 | 0.8 | 5% | 2% | 29% | 13% | 3% |
| 384 | Skåne | 0.8 | 0.8 | 9% | 7% | 10% | 15% | 8% |
| 385 | Skåne | 0.8 | 0.8 | 11% | 6% | 9% | 14% | 7% |
| 386 | Skåne | 0.8 | 0.8 | 11% | 3% | 19% | 10% | 5% |
| 387 | Skåne | 0.8 | 0.7 | 13% | 3% | 13% | 13% | 4% |
| 388 | Skåne | 0.8 | 0.7 | 12% | 3% | 14% | 13% | 6% |
| 389 | Skåne | 0.8 | 0.7 | 10% | 5% | 11% | 14% | 7% |
| 390 | Skåne | 0.8 | 0.7 | 8% | 5% | 15% | 13% | 7% |
| 391 | Skåne | 0.8 | 0.7 | 8% | 6% | 9% | 15% | 10% |
| 392 | Skåne | 0.8 | 0.7 | 13% | 3% | 13% | 12% | 4% |
| 393 | Skåne | 0.8 | 0.7 | 10% | 5% | 13% | 12% | 5% |
| 394 | Skåne | 0.8 | 0.7 | 11% | 5% | 11% | 12% | 6% |
| 395 | Skåne | 0.8 | 0.7 | 11% | 4% | 8% | 13% | 9% |
| 396 | Skåne | 0.8 | 0.7 | 12% | 5% | 17% | 9% | 3% |
| 397 | Skåne | 0.8 | 0.7 | 7% | 6% | 14% | 12% | 6% |
| 398 | Skåne | 0.7 | 0.7 | 4% | 5% | 15% | 13% | 6% |
| 399 | Skåne | 0.7 | 0.7 | 8% | 3% | 19% | 8% | 4% |
| 400 | Skåne | 0.7 | 0.7 | 8% | 4% | 13% | 10% | 6% |
| 401 | Skåne | 0.7 | 0.6 | 5% | 7% | 13% | 12% | 4% |
| 402 | Skåne | 0.7 | 0.6 | 7% | 4% | 14% | 11% | 4% |
| 403 | Skåne | 0.7 | 0.6 | 5% | 5% | 14% | 10% | 7% |
| 404 | Skåne | 0.7 | 0.6 | 7% | 3% | 15% | 11% | 3% |
| 405 | Skåne | 0.7 | 0.6 | 7% | 5% | 13% | 9% | 6% |
| 406 | Skåne | 0.7 | 0.6 | 7% | 5% | 10% | 10% | 7% |
| 407 | Skåne | 0.7 | 0.6 | 4% | 7% | 11% | 11% | 7% |
| 408 | Skåne | 0.7 | 0.6 | 7% | 6% | 13% | 9% | 4% |
| 409 | Skåne | 0.6 | 0.6 | 9% | 4% | 18% | 4% | 3% |
| 410 | Skåne | 0.6 | 0.6 | 8% | 3% | 15% | 7% | 4% |
| 411 | Skåne | 0.6 | 0.6 | 2% | 5% | 15% | 10% | 7% |
| 412 | Skåne | 0.6 | 0.6 | 4% | 5% | 12% | 10% | 8% |
| 413 | Skåne | 0.6 | 0.6 | 6% | 4% | 11% | 12% | 5% |
| 414 | Skåne | 0.6 | 0.6 | 4% | 4% | 17% | 8% | 4% |
| 415 | Skåne | 0.6 | 0.6 | 4% | 5% | 9% | 9% | 10% |
| 416 | Skåne | 0.6 | 0.5 | 5% | 3% | 21% | 7% | 1% |
| 417 | Skåne | 0.6 | 0.5 | 6% | 5% | 11% | 8% | 4% |
| 418 | Skåne | 0.6 | 0.5 | 6% | 3% | 11% | 8% | 4% |
| 419 | Skåne | 0.5 | 0.5 | 4% | 4% | 12% | 6% | 5% |
| 420 | Skåne | 0.5 | 0.5 | 9% | 2% | 7% | 7% | 4% |
| 421 | Skåne | 0.5 | 0.5 | 4% | 2% | 11% | 7% | 6% |
| 422 | Skåne | 0.5 | 0.4 | 3% | 4% | 9% | 9% | 4% |
| 423 | Skåne | 0.5 | 0.4 | 3% | 3% | 14% | 7% | 2% |
| 424 | Skåne | 0.5 | 0.4 | 7% | 3% | 8% | 6% | 3% |
| 425 | Skåne | 0.5 | 0.4 | 3% | 3% | 14% | 6% | 3% |
| 426 | Skåne | 0.5 | 0.4 | 6% | 4% | 8% | 7% | 2% |
| 427 | Skåne | 0.4 | 0.4 | 4% | 2% | 11% | 5% | 2% |
| 428 | Stockholm | 3.5 | 3.7 | 82% | 18% | 16% | 43% | 31% |
| 429 | Stockholm | 3.3 | 3.5 | 81% | 11% | 22% | 40% | 22% |
| 430 | Stockholm | 2.9 | 3.1 | 76% | 10% | 15% | 37% | 19% |
| 431 | Stockholm | 2.7 | 2.9 | 64% | 12% | 20% | 32% | 16% |
| 432 | Stockholm | 2.7 | 2.8 | 76% | 9% | 9% | 30% | 17% |
| 433 | Stockholm | 2.7 | 2.8 | 71% | 10% | 18% | 29% | 14% |
| 434 | Stockholm | 2.6 | 2.7 | 63% | 11% | 13% | 32% | 20% |
| 435 | Stockholm | 2.5 | 2.7 | 62% | 5% | 17% | 33% | 21% |
| 436 | Stockholm | 2.5 | 2.6 | 55% | 10% | 16% | 33% | 20% |
| 437 | Stockholm | 2.4 | 2.6 | 63% | 8% | 19% | 29% | 11% |
| 438 | Stockholm | 2.4 | 2.6 | 58% | 10% | 14% | 29% | 18% |
| 439 | Stockholm | 2.4 | 2.5 | 57% | 13% | 13% | 29% | 15% |
| 440 | Stockholm | 2.3 | 2.5 | 55% | 11% | 14% | 30% | 15% |
| 441 | Stockholm | 2.3 | 2.5 | 57% | 8% | 17% | 28% | 13% |
| 442 | Stockholm | 2.3 | 2.4 | 56% | 8% | 12% | 29% | 18% |
| 443 | Stockholm | 2.3 | 2.4 | 53% | 9% | 20% | 29% | 12% |
| 444 | Stockholm | 2.2 | 2.4 | 56% | 11% | 15% | 24% | 13% |
| 445 | Stockholm | 2.2 | 2.3 | 56% | 11% | 14% | 25% | 11% |
| 446 | Stockholm | 2.1 | 2.2 | 51% | 7% | 17% | 24% | 14% |
| 447 | Stockholm | 2.0 | 2.1 | 48% | 9% | 14% | 23% | 12% |
| 448 | Stockholm | 1.9 | 2.0 | 43% | 11% | 10% | 24% | 16% |
| 449 | Stockholm | 1.7 | 1.8 | 25% | 9% | 27% | 22% | 14% |
| 450 | Stockholm | 1.7 | 1.8 | 38% | 10% | 15% | 19% | 9% |
| 451 | Stockholm | 1.6 | 1.7 | 25% | 10% | 12% | 28% | 18% |
| 452 | Stockholm | 1.6 | 1.7 | 38% | 8% | 17% | 17% | 8% |
| 453 | Stockholm | 1.6 | 1.7 | 37% | 7% | 15% | 18% | 9% |
| 454 | Stockholm | 1.6 | 1.7 | 34% | 8% | 14% | 19% | 11% |
| 455 | Stockholm | 1.5 | 1.6 | 34% | 6% | 18% | 17% | 7% |
| 456 | Stockholm | 1.5 | 1.6 | 30% | 9% | 15% | 18% | 8% |
| 457 | Stockholm | 1.5 | 1.5 | 34% | 6% | 12% | 16% | 12% |
| 458 | Stockholm | 1.4 | 1.5 | 33% | 6% | 14% | 16% | 8% |
| 459 | Stockholm | 1.4 | 1.5 | 28% | 8% | 15% | 16% | 11% |
| 460 | Stockholm | 1.4 | 1.5 | 26% | 8% | 14% | 18% | 11% |
| 461 | Stockholm | 1.3 | 1.4 | 31% | 6% | 17% | 13% | 5% |
| 462 | Stockholm | 1.3 | 1.3 | 26% | 5% | 15% | 15% | 8% |
| 463 | Stockholm | 1.3 | 1.3 | 22% | 9% | 16% | 16% | 7% |
| 464 | Stockholm | 1.3 | 1.3 | 32% | 6% | 16% | 11% | 3% |
| 465 | Stockholm | 1.2 | 1.3 | 23% | 8% | 15% | 15% | 8% |
| 466 | Stockholm | 1.2 | 1.3 | 24% | 8% | 16% | 14% | 6% |
| 467 | Stockholm | 1.2 | 1.3 | 16% | 8% | 15% | 18% | 12% |
| 468 | Stockholm | 1.2 | 1.3 | 27% | 5% | 15% | 12% | 7% |
| 469 | Stockholm | 1.1 | 1.2 | 21% | 6% | 17% | 12% | 8% |
| 470 | Stockholm | 1.1 | 1.2 | 20% | 6% | 24% | 12% | 2% |
| 471 | Stockholm | 1.1 | 1.2 | 17% | 6% | 15% | 14% | 11% |
| 472 | Stockholm | 1.1 | 1.2 | 21% | 6% | 16% | 12% | 6% |
| 473 | Stockholm | 1.1 | 1.2 | 15% | 6% | 14% | 16% | 12% |
| 474 | Stockholm | 1.1 | 1.1 | 16% | 6% | 20% | 13% | 7% |
| 475 | Stockholm | 1.1 | 1.1 | 16% | 6% | 22% | 14% | 3% |
| 476 | Stockholm | 1.0 | 1.1 | 20% | 6% | 16% | 10% | 6% |
| 477 | Stockholm | 1.0 | 1.1 | 17% | 7% | 11% | 13% | 9% |
| 478 | Stockholm | 1.0 | 1.1 | 18% | 6% | 11% | 12% | 10% |
| 479 | Stockholm | 1.0 | 1.1 | 16% | 9% | 12% | 14% | 5% |
| 480 | Stockholm | 1.0 | 1.1 | 11% | 8% | 11% | 16% | 12% |
| 481 | Stockholm | 1.0 | 1.0 | 17% | 7% | 15% | 12% | 5% |
| 482 | Stockholm | 1.0 | 1.0 | 18% | 7% | 16% | 10% | 5% |
| 483 | Stockholm | 1.0 | 1.0 | 18% | 6% | 15% | 10% | 6% |
| 484 | Stockholm | 1.0 | 1.0 | 13% | 7% | 15% | 11% | 9% |
| 485 | Stockholm | 1.0 | 1.0 | 18% | 6% | 12% | 11% | 6% |
| 486 | Stockholm | 0.9 | 1.0 | 15% | 7% | 15% | 11% | 4% |
| 487 | Stockholm | 0.9 | 1.0 | 9% | 8% | 14% | 13% | 9% |
| 488 | Stockholm | 0.9 | 0.9 | 14% | 6% | 13% | 10% | 7% |
| 489 | Stockholm | 0.9 | 0.9 | 16% | 5% | 14% | 9% | 6% |
| 490 | Stockholm | 0.9 | 0.9 | 18% | 4% | 15% | 8% | 3% |
| 491 | Stockholm | 0.9 | 0.9 | 6% | 5% | 29% | 9% | 5% |
| 492 | Stockholm | 0.8 | 0.9 | 15% | 6% | 13% | 9% | 5% |
| 493 | Stockholm | 0.8 | 0.9 | 14% | 6% | 17% | 8% | 3% |
| 494 | Stockholm | 0.8 | 0.9 | 5% | 7% | 20% | 13% | 5% |
| 495 | Stockholm | 0.8 | 0.9 | 15% | 5% | 18% | 8% | 3% |
| 496 | Stockholm | 0.8 | 0.9 | 10% | 8% | 14% | 12% | 4% |
| 497 | Stockholm | 0.8 | 0.9 | 13% | 4% | 17% | 9% | 5% |
| 498 | Stockholm | 0.8 | 0.9 | 12% | 6% | 14% | 10% | 4% |
| 499 | Stockholm | 0.8 | 0.9 | 10% | 5% | 15% | 9% | 8% |
| 500 | Stockholm | 0.8 | 0.9 | 10% | 4% | 12% | 10% | 9% |
| 501 | Stockholm | 0.8 | 0.8 | 11% | 5% | 19% | 8% | 2% |
| 502 | Stockholm | 0.8 | 0.8 | 13% | 4% | 17% | 8% | 3% |
| 503 | Stockholm | 0.8 | 0.8 | 14% | 5% | 16% | 8% | 3% |
| 504 | Stockholm | 0.8 | 0.8 | 11% | 5% | 15% | 8% | 6% |
| 505 | Stockholm | 0.8 | 0.8 | 13% | 6% | 15% | 8% | 2% |
| 506 | Stockholm | 0.8 | 0.8 | 9% | 8% | 18% | 8% | 3% |
| 507 | Stockholm | 0.8 | 0.8 | 8% | 5% | 17% | 9% | 6% |
| 508 | Stockholm | 0.8 | 0.8 | 8% | 7% | 18% | 10% | 2% |
| 509 | Stockholm | 0.8 | 0.8 | 12% | 4% | 18% | 7% | 3% |
| 510 | Stockholm | 0.8 | 0.8 | 10% | 7% | 13% | 10% | 3% |
| 511 | Stockholm | 0.7 | 0.8 | 11% | 4% | 14% | 8% | 6% |
| 512 | Stockholm | 0.7 | 0.8 | 7% | 6% | 13% | 10% | 8% |
| 513 | Stockholm | 0.7 | 0.8 | 9% | 6% | 14% | 10% | 3% |
| 514 | Stockholm | 0.7 | 0.8 | 8% | 7% | 14% | 10% | 3% |
| 515 | Stockholm | 0.7 | 0.8 | 12% | 5% | 11% | 9% | 3% |
| 516 | Stockholm | 0.7 | 0.8 | 9% | 6% | 16% | 8% | 3% |
| 517 | Stockholm | 0.7 | 0.8 | 10% | 5% | 18% | 7% | 2% |
| 518 | Stockholm | 0.7 | 0.8 | 10% | 5% | 19% | 6% | 2% |
| 519 | Stockholm | 0.7 | 0.8 | 9% | 5% | 17% | 7% | 3% |
| 520 | Stockholm | 0.7 | 0.7 | 11% | 5% | 16% | 6% | 2% |
| 521 | Stockholm | 0.7 | 0.7 | 10% | 5% | 17% | 6% | 2% |
| 522 | Stockholm | 0.7 | 0.7 | 10% | 6% | 14% | 8% | 2% |
| 523 | Stockholm | 0.7 | 0.7 | 12% | 2% | 18% | 5% | 2% |
| 524 | Stockholm | 0.7 | 0.7 | 8% | 6% | 17% | 7% | 2% |
| 525 | Stockholm | 0.7 | 0.7 | 9% | 5% | 17% | 6% | 3% |
| 526 | Stockholm | 0.7 | 0.7 | 8% | 5% | 16% | 8% | 3% |
| 527 | Stockholm | 0.7 | 0.7 | 7% | 3% | 26% | 3% | 2% |
| 528 | Stockholm | 0.7 | 0.7 | 8% | 5% | 16% | 6% | 5% |
| 529 | Stockholm | 0.7 | 0.7 | 9% | 6% | 14% | 8% | 2% |
| 530 | Stockholm | 0.7 | 0.7 | 6% | 4% | 16% | 8% | 6% |
| 531 | Stockholm | 0.7 | 0.7 | 6% | 6% | 15% | 9% | 2% |
| 532 | Stockholm | 0.6 | 0.7 | 6% | 6% | 17% | 7% | 1% |
| 533 | Stockholm | 0.6 | 0.7 | 8% | 4% | 15% | 7% | 2% |
| 534 | Stockholm | 0.6 | 0.7 | 4% | 5% | 21% | 5% | 2% |
| 535 | Stockholm | 0.6 | 0.7 | 9% | 3% | 15% | 5% | 4% |
| 536 | Stockholm | 0.6 | 0.7 | 7% | 3% | 20% | 6% | 2% |
| 537 | Stockholm | 0.6 | 0.6 | 7% | 6% | 12% | 9% | 1% |
| 538 | Stockholm | 0.6 | 0.6 | 7% | 4% | 13% | 7% | 5% |
| 539 | Stockholm | 0.6 | 0.6 | 7% | 4% | 15% | 5% | 2% |
| 540 | Stockholm | 0.6 | 0.6 | 5% | 5% | 14% | 7% | 3% |
| 541 | Stockholm | 0.6 | 0.6 | 10% | 2% | 14% | 5% | 3% |
| 542 | Stockholm | 0.6 | 0.6 | 7% | 5% | 14% | 5% | 2% |
| 543 | Stockholm | 0.6 | 0.6 | 7% | 5% | 15% | 5% | 3% |
| 544 | Stockholm | 0.6 | 0.6 | 4% | 5% | 14% | 8% | 3% |
| 545 | Södermanland | 3.2 | 2.2 | 68% | 13% | 11% | 50% | 29% |
| 546 | Södermanland | 2.9 | 2.0 | 60% | 10% | 5% | 50% | 32% |
| 547 | Södermanland | 2.5 | 1.7 | 52% | 12% | 12% | 38% | 18% |
| 548 | Södermanland | 2.3 | 1.6 | 45% | 9% | 23% | 36% | 14% |
| 549 | Södermanland | 2.3 | 1.6 | 49% | 12% | 14% | 33% | 17% |
| 550 | Södermanland | 2.0 | 1.4 | 37% | 12% | 13% | 26% | 22% |
| 551 | Södermanland | 1.9 | 1.3 | 36% | 10% | 13% | 30% | 16% |
| 552 | Södermanland | 1.8 | 1.3 | 33% | 9% | 11% | 30% | 17% |
| 553 | Södermanland | 1.8 | 1.3 | 33% | 10% | 12% | 28% | 18% |
| 554 | Södermanland | 1.8 | 1.2 | 35% | 8% | 8% | 29% | 16% |
| 555 | Södermanland | 1.7 | 1.2 | 27% | 11% | 13% | 25% | 18% |
| 556 | Södermanland | 1.6 | 1.1 | 28% | 9% | 13% | 23% | 16% |
| 557 | Södermanland | 1.6 | 1.1 | 28% | 9% | 13% | 26% | 10% |
| 558 | Södermanland | 1.5 | 1.0 | 25% | 8% | 13% | 25% | 12% |
| 559 | Södermanland | 1.4 | 1.0 | 21% | 8% | 24% | 20% | 9% |
| 560 | Södermanland | 1.4 | 1.0 | 18% | 10% | 14% | 22% | 17% |
| 561 | Södermanland | 1.4 | 1.0 | 21% | 11% | 9% | 22% | 16% |
| 562 | Södermanland | 1.3 | 0.9 | 22% | 8% | 15% | 18% | 10% |
| 563 | Södermanland | 1.3 | 0.9 | 18% | 8% | 12% | 22% | 11% |
| 564 | Södermanland | 1.2 | 0.8 | 22% | 6% | 12% | 16% | 9% |
| 565 | Södermanland | 1.1 | 0.8 | 17% | 7% | 12% | 18% | 11% |
| 566 | Södermanland | 1.1 | 0.8 | 14% | 8% | 18% | 17% | 9% |
| 567 | Södermanland | 1.1 | 0.7 | 14% | 7% | 16% | 16% | 8% |
| 568 | Södermanland | 0.9 | 0.6 | 10% | 8% | 12% | 13% | 12% |
| 569 | Södermanland | 0.9 | 0.6 | 13% | 5% | 15% | 13% | 5% |
| 570 | Södermanland | 0.9 | 0.6 | 13% | 6% | 11% | 13% | 6% |
| 571 | Södermanland | 0.9 | 0.6 | 10% | 6% | 15% | 9% | 10% |
| 572 | Södermanland | 0.8 | 0.6 | 6% | 5% | 21% | 9% | 7% |
| 573 | Södermanland | 0.8 | 0.5 | 4% | 8% | 12% | 12% | 12% |
| 574 | Uppsala | 2.6 | 2.6 | 62% | 11% | 13% | 37% | 16% |
| 575 | Uppsala | 2.4 | 2.4 | 54% | 8% | 16% | 34% | 15% |
| 576 | Uppsala | 2.0 | 2.0 | 45% | 9% | 18% | 25% | 11% |
| 577 | Uppsala | 1.8 | 1.8 | 31% | 9% | 17% | 27% | 14% |
| 578 | Uppsala | 1.7 | 1.7 | 34% | 9% | 11% | 26% | 14% |
| 579 | Uppsala | 1.6 | 1.6 | 35% | 8% | 16% | 19% | 10% |
| 580 | Uppsala | 1.6 | 1.6 | 28% | 10% | 8% | 25% | 16% |
| 581 | Uppsala | 1.5 | 1.5 | 23% | 8% | 14% | 25% | 15% |
| 582 | Uppsala | 1.4 | 1.4 | 24% | 8% | 11% | 22% | 15% |
| 583 | Uppsala | 1.4 | 1.4 | 30% | 7% | 12% | 20% | 6% |
| 584 | Uppsala | 1.4 | 1.3 | 29% | 4% | 23% | 15% | 3% |
| 585 | Uppsala | 1.3 | 1.3 | 19% | 8% | 22% | 14% | 12% |
| 586 | Uppsala | 1.3 | 1.2 | 28% | 5% | 11% | 16% | 7% |
| 587 | Uppsala | 1.2 | 1.2 | 13% | 7% | 17% | 21% | 11% |
| 588 | Uppsala | 1.2 | 1.2 | 18% | 5% | 30% | 11% | 3% |
| 589 | Uppsala | 1.1 | 1.1 | 22% | 4% | 19% | 14% | 3% |
| 590 | Uppsala | 1.1 | 1.1 | 18% | 6% | 18% | 16% | 7% |
| 591 | Uppsala | 1.1 | 1.1 | 20% | 6% | 14% | 15% | 7% |
| 592 | Uppsala | 1.1 | 1.1 | 15% | 7% | 11% | 18% | 13% |
| 593 | Uppsala | 1.1 | 1.1 | 20% | 5% | 16% | 13% | 6% |
| 594 | Uppsala | 1.0 | 1.0 | 7% | 8% | 14% | 17% | 13% |
| 595 | Uppsala | 1.0 | 1.0 | 7% | 7% | 18% | 18% | 8% |
| 596 | Uppsala | 0.9 | 0.9 | 16% | 6% | 14% | 10% | 4% |
| 597 | Uppsala | 0.9 | 0.9 | 14% | 5% | 16% | 11% | 4% |
| 598 | Uppsala | 0.9 | 0.9 | 14% | 4% | 15% | 11% | 6% |
| 599 | Uppsala | 0.9 | 0.8 | 15% | 6% | 12% | 11% | 4% |
| 600 | Uppsala | 0.8 | 0.8 | 5% | 10% | 14% | 11% | 11% |
| 601 | Uppsala | 0.8 | 0.8 | 16% | 5% | 14% | 8% | 4% |
| 602 | Uppsala | 0.8 | 0.8 | 7% | 8% | 9% | 15% | 7% |
| 603 | Uppsala | 0.8 | 0.8 | 10% | 6% | 12% | 11% | 7% |
| 604 | Uppsala | 0.8 | 0.8 | 6% | 8% | 10% | 11% | 10% |
| 605 | Uppsala | 0.8 | 0.8 | 7% | 4% | 21% | 8% | 4% |
| 606 | Uppsala | 0.8 | 0.7 | 13% | 5% | 13% | 9% | 3% |
| 607 | Uppsala | 0.8 | 0.7 | 7% | 7% | 11% | 7% | 12% |
| 608 | Uppsala | 0.7 | 0.7 | 11% | 5% | 13% | 8% | 5% |
| 609 | Uppsala | 0.7 | 0.7 | 10% | 5% | 15% | 8% | 5% |
| 610 | Uppsala | 0.7 | 0.7 | 10% | 4% | 11% | 10% | 7% |
| 611 | Uppsala | 0.7 | 0.7 | 6% | 5% | 14% | 8% | 8% |
| 612 | Uppsala | 0.6 | 0.6 | 4% | 3% | 16% | 8% | 8% |
| 613 | Uppsala | 0.6 | 0.6 | 4% | 4% | 9% | 8% | 8% |
| 614 | Uppsala | 0.5 | 0.5 | 4% | 6% | 9% | 5% | 8% |
| 615 | Uppsala | 0.5 | 0.5 | 2% | 5% | 10% | 7% | 6% |
| 616 | Uppsala | 0.5 | 0.5 | 5% | 1% | 10% | 7% | 4% |
| 617 | Uppsala | 0.4 | 0.4 | 4% | 2% | 9% | 5% | 4% |
| 618 | Värmland | 1.9 | 2.2 | 41% | 6% | 10% | 32% | 14% |
| 619 | Värmland | 1.8 | 2.1 | 34% | 7% | 15% | 30% | 13% |
| 620 | Värmland | 1.6 | 1.8 | 27% | 9% | 9% | 26% | 19% |
| 621 | Värmland | 1.4 | 1.5 | 13% | 8% | 13% | 30% | 14% |
| 622 | Värmland | 1.3 | 1.5 | 19% | 8% | 7% | 27% | 12% |
| 623 | Värmland | 1.3 | 1.4 | 19% | 7% | 7% | 26% | 11% |
| 624 | Värmland | 1.2 | 1.4 | 20% | 7% | 12% | 21% | 8% |
| 625 | Värmland | 1.2 | 1.3 | 17% | 7% | 7% | 24% | 10% |
| 626 | Värmland | 1.2 | 1.3 | 13% | 9% | 9% | 21% | 14% |
| 627 | Värmland | 1.0 | 1.2 | 12% | 8% | 10% | 19% | 9% |
| 628 | Värmland | 1.0 | 1.1 | 15% | 6% | 4% | 17% | 11% |
| 629 | Värmland | 0.9 | 1.0 | 9% | 7% | 7% | 20% | 8% |
| 630 | Värmland | 0.9 | 1.0 | 13% | 5% | 12% | 14% | 5% |
| 631 | Värmland | 0.9 | 1.0 | 10% | 6% | 5% | 17% | 10% |
| 632 | Värmland | 0.8 | 1.0 | 9% | 7% | 11% | 15% | 6% |
| 633 | Värmland | 0.8 | 1.0 | 10% | 7% | 6% | 16% | 9% |
| 634 | Värmland | 0.8 | 0.9 | 7% | 10% | 6% | 17% | 7% |
| 635 | Värmland | 0.7 | 0.8 | 7% | 5% | 10% | 13% | 6% |
| 636 | Värmland | 0.7 | 0.8 | 6% | 6% | 18% | 10% | 2% |
| 637 | Värmland | 0.7 | 0.8 | 6% | 9% | 9% | 14% | 4% |
| 638 | Värmland | 0.7 | 0.8 | 1% | 8% | 4% | 16% | 14% |
| 639 | Värmland | 0.7 | 0.8 | 6% | 6% | 6% | 14% | 8% |
| 640 | Värmland | 0.7 | 0.7 | 6% | 6% | 15% | 9% | 4% |
| 641 | Värmland | 0.5 | 0.5 | 5% | 4% | 8% | 8% | 3% |
| 642 | Värmland | 0.4 | 0.4 | 3% | 3% | 9% | 6% | 2% |
| 643 | Västernorrland | 2.3 | 2.3 | 43% | 9% | 11% | 39% | 20% |
| 644 | Västernorrland | 2.2 | 2.3 | 42% | 7% | 13% | 39% | 19% |
| 645 | Västernorrland | 1.8 | 1.9 | 30% | 10% | 18% | 28% | 15% |
| 646 | Västernorrland | 1.7 | 1.8 | 28% | 10% | 10% | 29% | 17% |
| 647 | Västernorrland | 1.7 | 1.7 | 32% | 10% | 11% | 26% | 13% |
| 648 | Västernorrland | 1.6 | 1.7 | 24% | 10% | 11% | 30% | 15% |
| 649 | Västernorrland | 1.5 | 1.6 | 25% | 8% | 10% | 28% | 12% |
| 650 | Västernorrland | 1.5 | 1.5 | 21% | 9% | 11% | 26% | 15% |
| 651 | Västernorrland | 1.4 | 1.5 | 22% | 9% | 17% | 21% | 11% |
| 652 | Västernorrland | 1.3 | 1.3 | 21% | 6% | 11% | 22% | 9% |
| 653 | Västernorrland | 1.2 | 1.3 | 16% | 8% | 11% | 21% | 14% |
| 654 | Västernorrland | 1.0 | 1.1 | 15% | 5% | 8% | 20% | 9% |
| 655 | Västernorrland | 1.0 | 1.1 | 9% | 10% | 9% | 20% | 9% |
| 656 | Västernorrland | 1.0 | 1.0 | 14% | 7% | 13% | 16% | 8% |
| 657 | Västernorrland | 1.0 | 1.0 | 12% | 7% | 9% | 19% | 8% |
| 658 | Västernorrland | 1.0 | 1.0 | 12% | 7% | 10% | 19% | 6% |
| 659 | Västernorrland | 0.9 | 1.0 | 6% | 7% | 11% | 18% | 13% |
| 660 | Västernorrland | 0.9 | 0.9 | 10% | 6% | 10% | 17% | 7% |
| 661 | Västernorrland | 0.8 | 0.9 | 5% | 5% | 10% | 19% | 10% |
| 662 | Västernorrland | 0.8 | 0.9 | 6% | 5% | 11% | 16% | 9% |
| 663 | Västernorrland | 0.8 | 0.9 | 4% | 7% | 11% | 17% | 9% |
| 664 | Västernorrland | 0.8 | 0.8 | 8% | 6% | 10% | 13% | 7% |
| 665 | Västernorrland | 0.8 | 0.8 | 5% | 5% | 11% | 16% | 8% |
| 666 | Västernorrland | 0.7 | 0.8 | 5% | 7% | 11% | 11% | 10% |
| 667 | Västernorrland | 0.7 | 0.7 | 7% | 5% | 8% | 13% | 7% |
| 668 | Västernorrland | 0.7 | 0.7 | 6% | 5% | 11% | 10% | 8% |
| 669 | Västernorrland | 0.7 | 0.7 | 6% | 6% | 13% | 10% | 5% |
| 670 | Västernorrland | 0.7 | 0.7 | 7% | 3% | 7% | 14% | 7% |
| 671 | Västernorrland | 0.6 | 0.7 | 5% | 5% | 8% | 13% | 7% |
| 672 | Västernorrland | 0.6 | 0.6 | 5% | 4% | 9% | 12% | 3% |
| 673 | Västernorrland | 0.5 | 0.5 | 6% | 3% | 7% | 10% | 4% |
| 674 | Västmanland | 3.5 | 2.5 | 80% | 14% | 21% | 47% | 25% |
| 675 | Västmanland | 2.9 | 2.1 | 65% | 11% | 16% | 38% | 25% |
| 676 | Västmanland | 2.7 | 2.0 | 64% | 10% | 12% | 39% | 21% |
| 677 | Västmanland | 2.4 | 1.8 | 52% | 11% | 10% | 35% | 23% |
| 678 | Västmanland | 2.4 | 1.7 | 50% | 11% | 16% | 33% | 19% |
| 679 | Västmanland | 1.7 | 1.3 | 32% | 11% | 11% | 26% | 18% |
| 680 | Västmanland | 1.7 | 1.3 | 34% | 10% | 7% | 27% | 17% |
| 681 | Västmanland | 1.6 | 1.2 | 22% | 12% | 10% | 29% | 18% |
| 682 | Västmanland | 1.6 | 1.2 | 31% | 8% | 16% | 21% | 11% |
| 683 | Västmanland | 1.6 | 1.1 | 29% | 8% | 7% | 27% | 14% |
| 684 | Västmanland | 1.5 | 1.1 | 29% | 8% | 15% | 22% | 10% |
| 685 | Västmanland | 1.5 | 1.1 | 27% | 8% | 7% | 24% | 14% |
| 686 | Västmanland | 1.4 | 1.0 | 22% | 8% | 13% | 21% | 15% |
| 687 | Västmanland | 1.4 | 1.0 | 24% | 7% | 9% | 23% | 16% |
| 688 | Västmanland | 1.4 | 1.0 | 19% | 11% | 7% | 23% | 18% |
| 689 | Västmanland | 1.3 | 1.0 | 24% | 7% | 18% | 18% | 8% |
| 690 | Västmanland | 1.2 | 0.9 | 22% | 8% | 13% | 17% | 10% |
| 691 | Västmanland | 1.2 | 0.9 | 20% | 8% | 9% | 20% | 11% |
| 692 | Västmanland | 1.2 | 0.9 | 16% | 8% | 13% | 20% | 10% |
| 693 | Västmanland | 1.2 | 0.8 | 18% | 7% | 9% | 19% | 12% |
| 694 | Västmanland | 1.1 | 0.8 | 21% | 6% | 15% | 14% | 7% |
| 695 | Västmanland | 1.1 | 0.8 | 21% | 5% | 15% | 16% | 4% |
| 696 | Västmanland | 1.1 | 0.8 | 16% | 7% | 17% | 14% | 9% |
| 697 | Västmanland | 1.1 | 0.8 | 18% | 6% | 13% | 14% | 8% |
| 698 | Västmanland | 1.0 | 0.7 | 11% | 7% | 13% | 16% | 11% |
| 699 | Västmanland | 0.9 | 0.7 | 14% | 6% | 15% | 12% | 7% |
| 700 | Västmanland | 0.8 | 0.6 | 8% | 10% | 12% | 11% | 10% |
| 701 | Västmanland | 0.8 | 0.6 | 11% | 5% | 17% | 9% | 5% |
| 702 | Västmanland | 0.8 | 0.6 | 3% | 10% | 15% | 13% | 8% |
| 703 | Västra Götaland | 3.3 | 3.4 | 67% | 13% | 17% | 53% | 33% |
| 704 | Västra Götaland | 3.1 | 3.2 | 70% | 15% | 16% | 42% | 25% |
| 705 | Västra Götaland | 3.1 | 3.2 | 69% | 14% | 19% | 43% | 25% |
| 706 | Västra Götaland | 2.9 | 3.0 | 67% | 13% | 17% | 39% | 22% |
| 707 | Västra Götaland | 2.9 | 2.9 | 61% | 11% | 12% | 47% | 23% |
| 708 | Västra Götaland | 2.8 | 2.9 | 62% | 10% | 16% | 42% | 23% |
| 709 | Västra Götaland | 2.8 | 2.9 | 63% | 15% | 18% | 38% | 19% |
| 710 | Västra Götaland | 2.8 | 2.9 | 64% | 8% | 19% | 42% | 18% |
| 711 | Västra Götaland | 2.8 | 2.8 | 65% | 7% | 33% | 36% | 9% |
| 712 | Västra Götaland | 2.8 | 2.8 | 59% | 15% | 18% | 39% | 22% |
| 713 | Västra Götaland | 2.8 | 2.8 | 57% | 10% | 18% | 42% | 25% |
| 714 | Västra Götaland | 2.8 | 2.8 | 64% | 9% | 19% | 40% | 17% |
| 715 | Västra Götaland | 2.8 | 2.8 | 58% | 14% | 16% | 39% | 22% |
| 716 | Västra Götaland | 2.7 | 2.8 | 62% | 12% | 13% | 37% | 24% |
| 717 | Västra Götaland | 2.7 | 2.8 | 60% | 12% | 15% | 39% | 22% |
| 718 | Västra Götaland | 2.5 | 2.6 | 60% | 6% | 14% | 36% | 21% |
| 719 | Västra Götaland | 2.5 | 2.6 | 56% | 11% | 16% | 35% | 19% |
| 720 | Västra Götaland | 2.4 | 2.5 | 49% | 9% | 13% | 42% | 19% |
| 721 | Västra Götaland | 2.4 | 2.4 | 42% | 11% | 13% | 40% | 24% |
| 722 | Västra Götaland | 2.3 | 2.4 | 51% | 12% | 23% | 27% | 13% |
| 723 | Västra Götaland | 2.3 | 2.3 | 43% | 11% | 10% | 40% | 22% |
| 724 | Västra Götaland | 2.3 | 2.3 | 51% | 10% | 20% | 27% | 15% |
| 725 | Västra Götaland | 2.2 | 2.2 | 43% | 8% | 13% | 34% | 20% |
| 726 | Västra Götaland | 2.2 | 2.2 | 40% | 11% | 27% | 30% | 13% |
| 727 | Västra Götaland | 2.0 | 2.1 | 42% | 9% | 17% | 30% | 14% |
| 728 | Västra Götaland | 2.0 | 2.1 | 45% | 10% | 19% | 26% | 10% |
| 729 | Västra Götaland | 2.0 | 2.1 | 34% | 8% | 16% | 34% | 21% |
| 730 | Västra Götaland | 2.0 | 2.0 | 48% | 8% | 20% | 24% | 8% |
| 731 | Västra Götaland | 2.0 | 2.0 | 40% | 10% | 22% | 25% | 10% |
| 732 | Västra Götaland | 1.9 | 2.0 | 38% | 8% | 16% | 29% | 15% |
| 733 | Västra Götaland | 1.9 | 2.0 | 39% | 7% | 12% | 28% | 19% |
| 734 | Västra Götaland | 1.9 | 1.9 | 38% | 12% | 24% | 23% | 9% |
| 735 | Västra Götaland | 1.8 | 1.9 | 40% | 10% | 17% | 23% | 12% |
| 736 | Västra Götaland | 1.8 | 1.8 | 31% | 9% | 14% | 30% | 14% |
| 737 | Västra Götaland | 1.7 | 1.7 | 34% | 8% | 16% | 23% | 11% |
| 738 | Västra Götaland | 1.7 | 1.7 | 33% | 10% | 15% | 23% | 11% |
| 739 | Västra Götaland | 1.7 | 1.7 | 32% | 9% | 22% | 22% | 8% |
| 740 | Västra Götaland | 1.7 | 1.7 | 29% | 9% | 11% | 28% | 15% |
| 741 | Västra Götaland | 1.6 | 1.6 | 26% | 9% | 10% | 29% | 14% |
| 742 | Västra Götaland | 1.6 | 1.6 | 26% | 8% | 15% | 27% | 11% |
| 743 | Västra Götaland | 1.5 | 1.6 | 31% | 5% | 23% | 16% | 9% |
| 744 | Västra Götaland | 1.5 | 1.5 | 24% | 9% | 15% | 23% | 11% |
| 745 | Västra Götaland | 1.4 | 1.5 | 29% | 8% | 18% | 16% | 8% |
| 746 | Västra Götaland | 1.4 | 1.5 | 26% | 6% | 23% | 19% | 6% |
| 747 | Västra Götaland | 1.4 | 1.5 | 27% | 6% | 13% | 23% | 9% |
| 748 | Västra Götaland | 1.4 | 1.4 | 24% | 7% | 28% | 15% | 6% |
| 749 | Västra Götaland | 1.4 | 1.4 | 19% | 6% | 11% | 28% | 14% |
| 750 | Västra Götaland | 1.4 | 1.4 | 26% | 7% | 14% | 19% | 11% |
| 751 | Västra Götaland | 1.4 | 1.4 | 24% | 8% | 9% | 21% | 15% |
| 752 | Västra Götaland | 1.4 | 1.4 | 20% | 7% | 8% | 26% | 14% |
| 753 | Västra Götaland | 1.3 | 1.4 | 22% | 7% | 11% | 25% | 10% |
| 754 | Västra Götaland | 1.3 | 1.4 | 19% | 8% | 13% | 23% | 13% |
| 755 | Västra Götaland | 1.3 | 1.4 | 20% | 8% | 7% | 27% | 11% |
| 756 | Västra Götaland | 1.3 | 1.3 | 23% | 8% | 21% | 15% | 5% |
| 757 | Västra Götaland | 1.3 | 1.3 | 27% | 7% | 15% | 16% | 7% |
| 758 | Västra Götaland | 1.3 | 1.3 | 22% | 5% | 5% | 24% | 15% |
| 759 | Västra Götaland | 1.3 | 1.3 | 24% | 6% | 20% | 13% | 7% |
| 760 | Västra Götaland | 1.3 | 1.3 | 17% | 8% | 13% | 20% | 13% |
| 761 | Västra Götaland | 1.3 | 1.3 | 23% | 6% | 18% | 14% | 9% |
| 762 | Västra Götaland | 1.2 | 1.3 | 21% | 8% | 18% | 18% | 5% |
| 763 | Västra Götaland | 1.2 | 1.3 | 24% | 5% | 14% | 16% | 10% |
| 764 | Västra Götaland | 1.2 | 1.3 | 21% | 6% | 11% | 21% | 9% |
| 765 | Västra Götaland | 1.2 | 1.2 | 14% | 8% | 16% | 20% | 13% |
| 766 | Västra Götaland | 1.2 | 1.2 | 25% | 4% | 14% | 16% | 6% |
| 767 | Västra Götaland | 1.2 | 1.2 | 20% | 5% | 21% | 13% | 8% |
| 768 | Västra Götaland | 1.2 | 1.2 | 23% | 6% | 14% | 16% | 7% |
| 769 | Västra Götaland | 1.2 | 1.2 | 20% | 9% | 19% | 16% | 2% |
| 770 | Västra Götaland | 1.2 | 1.2 | 19% | 7% | 10% | 19% | 12% |
| 771 | Västra Götaland | 1.2 | 1.2 | 16% | 8% | 12% | 19% | 12% |
| 772 | Västra Götaland | 1.2 | 1.2 | 18% | 7% | 15% | 16% | 9% |
| 773 | Västra Götaland | 1.2 | 1.2 | 18% | 4% | 26% | 13% | 5% |
| 774 | Västra Götaland | 1.2 | 1.2 | 20% | 8% | 18% | 14% | 5% |
| 775 | Västra Götaland | 1.2 | 1.2 | 16% | 8% | 10% | 19% | 13% |
| 776 | Västra Götaland | 1.2 | 1.2 | 17% | 4% | 33% | 10% | 2% |
| 777 | Västra Götaland | 1.1 | 1.2 | 16% | 6% | 11% | 20% | 11% |
| 778 | Västra Götaland | 1.1 | 1.2 | 20% | 6% | 11% | 17% | 9% |
| 779 | Västra Götaland | 1.1 | 1.2 | 19% | 5% | 9% | 19% | 11% |
| 780 | Västra Götaland | 1.1 | 1.2 | 17% | 6% | 11% | 17% | 13% |
| 781 | Västra Götaland | 1.1 | 1.1 | 23% | 6% | 14% | 14% | 6% |
| 782 | Västra Götaland | 1.1 | 1.1 | 10% | 6% | 16% | 20% | 13% |
| 783 | Västra Götaland | 1.1 | 1.1 | 24% | 6% | 16% | 11% | 4% |
| 784 | Västra Götaland | 1.1 | 1.1 | 18% | 6% | 9% | 19% | 9% |
| 785 | Västra Götaland | 1.1 | 1.1 | 17% | 7% | 12% | 16% | 11% |
| 786 | Västra Götaland | 1.1 | 1.1 | 15% | 7% | 12% | 17% | 11% |
| 787 | Västra Götaland | 1.1 | 1.1 | 15% | 7% | 10% | 18% | 12% |
| 788 | Västra Götaland | 1.1 | 1.1 | 17% | 6% | 15% | 15% | 8% |
| 789 | Västra Götaland | 1.1 | 1.1 | 19% | 4% | 22% | 11% | 4% |
| 790 | Västra Götaland | 1.1 | 1.1 | 19% | 6% | 16% | 12% | 7% |
| 791 | Västra Götaland | 1.1 | 1.1 | 21% | 6% | 17% | 11% | 5% |
| 792 | Västra Götaland | 1.1 | 1.1 | 19% | 10% | 17% | 9% | 5% |
| 793 | Västra Götaland | 1.1 | 1.1 | 18% | 2% | 25% | 11% | 4% |
| 794 | Västra Götaland | 1.0 | 1.1 | 25% | 5% | 12% | 11% | 4% |
| 795 | Västra Götaland | 1.0 | 1.1 | 14% | 7% | 19% | 14% | 5% |
| 796 | Västra Götaland | 1.0 | 1.1 | 14% | 5% | 8% | 18% | 13% |
| 797 | Västra Götaland | 1.0 | 1.0 | 20% | 6% | 15% | 12% | 4% |
| 798 | Västra Götaland | 1.0 | 1.0 | 15% | 6% | 12% | 16% | 9% |
| 799 | Västra Götaland | 1.0 | 1.0 | 15% | 6% | 21% | 12% | 4% |
| 800 | Västra Götaland | 1.0 | 1.0 | 16% | 7% | 20% | 11% | 3% |
| 801 | Västra Götaland | 1.0 | 1.0 | 15% | 6% | 11% | 15% | 10% |
| 802 | Västra Götaland | 1.0 | 1.0 | 18% | 6% | 17% | 11% | 4% |
| 803 | Västra Götaland | 1.0 | 1.0 | 13% | 5% | 17% | 16% | 6% |
| 804 | Västra Götaland | 1.0 | 1.0 | 15% | 5% | 15% | 13% | 7% |
| 805 | Västra Götaland | 1.0 | 1.0 | 19% | 5% | 12% | 12% | 7% |
| 806 | Västra Götaland | 1.0 | 1.0 | 18% | 6% | 11% | 15% | 5% |
| 807 | Västra Götaland | 1.0 | 1.0 | 15% | 5% | 17% | 14% | 3% |
| 808 | Västra Götaland | 1.0 | 1.0 | 16% | 9% | 15% | 11% | 4% |
| 809 | Västra Götaland | 1.0 | 1.0 | 10% | 8% | 15% | 15% | 9% |
| 810 | Västra Götaland | 1.0 | 1.0 | 13% | 9% | 12% | 15% | 6% |
| 811 | Västra Götaland | 1.0 | 1.0 | 12% | 8% | 8% | 19% | 8% |
| 812 | Västra Götaland | 1.0 | 1.0 | 9% | 8% | 12% | 16% | 10% |
| 813 | Västra Götaland | 0.9 | 1.0 | 13% | 7% | 23% | 9% | 3% |
| 814 | Västra Götaland | 0.9 | 1.0 | 12% | 8% | 7% | 14% | 13% |
| 815 | Västra Götaland | 0.9 | 1.0 | 9% | 4% | 11% | 16% | 15% |
| 816 | Västra Götaland | 0.9 | 1.0 | 12% | 7% | 13% | 15% | 6% |
| 817 | Västra Götaland | 0.9 | 1.0 | 17% | 5% | 16% | 10% | 4% |
| 818 | Västra Götaland | 0.9 | 1.0 | 14% | 6% | 15% | 11% | 6% |
| 819 | Västra Götaland | 0.9 | 0.9 | 15% | 5% | 20% | 10% | 3% |
| 820 | Västra Götaland | 0.9 | 0.9 | 17% | 5% | 16% | 9% | 5% |
| 821 | Västra Götaland | 0.9 | 0.9 | 11% | 6% | 11% | 16% | 8% |
| 822 | Västra Götaland | 0.9 | 0.9 | 16% | 6% | 12% | 11% | 7% |
| 823 | Västra Götaland | 0.9 | 0.9 | 11% | 8% | 15% | 14% | 4% |
| 824 | Västra Götaland | 0.9 | 0.9 | 15% | 5% | 17% | 11% | 3% |
| 825 | Västra Götaland | 0.9 | 0.9 | 8% | 6% | 12% | 15% | 11% |
| 826 | Västra Götaland | 0.9 | 0.9 | 12% | 5% | 11% | 13% | 10% |
| 827 | Västra Götaland | 0.9 | 0.9 | 11% | 6% | 11% | 15% | 7% |
| 828 | Västra Götaland | 0.9 | 0.9 | 10% | 7% | 7% | 16% | 9% |
| 829 | Västra Götaland | 0.9 | 0.9 | 10% | 6% | 12% | 14% | 8% |
| 830 | Västra Götaland | 0.9 | 0.9 | 11% | 5% | 11% | 15% | 8% |
| 831 | Västra Götaland | 0.9 | 0.9 | 10% | 7% | 19% | 11% | 2% |
| 832 | Västra Götaland | 0.8 | 0.9 | 11% | 5% | 12% | 12% | 8% |
| 833 | Västra Götaland | 0.8 | 0.9 | 10% | 7% | 17% | 12% | 3% |
| 834 | Västra Götaland | 0.8 | 0.9 | 6% | 6% | 17% | 14% | 8% |
| 835 | Västra Götaland | 0.8 | 0.9 | 12% | 6% | 9% | 13% | 8% |
| 836 | Västra Götaland | 0.8 | 0.9 | 12% | 6% | 12% | 11% | 8% |
| 837 | Västra Götaland | 0.8 | 0.9 | 9% | 6% | 12% | 13% | 8% |
| 838 | Västra Götaland | 0.8 | 0.8 | 7% | 8% | 15% | 14% | 6% |
| 839 | Västra Götaland | 0.8 | 0.8 | 13% | 6% | 20% | 7% | 2% |
| 840 | Västra Götaland | 0.8 | 0.8 | 12% | 4% | 16% | 10% | 5% |
| 841 | Västra Götaland | 0.8 | 0.8 | 9% | 7% | 8% | 14% | 8% |
| 842 | Västra Götaland | 0.8 | 0.8 | 13% | 5% | 19% | 7% | 3% |
| 843 | Västra Götaland | 0.8 | 0.8 | 9% | 6% | 18% | 12% | 2% |
| 844 | Västra Götaland | 0.8 | 0.8 | 10% | 6% | 14% | 11% | 6% |
| 845 | Västra Götaland | 0.8 | 0.8 | 8% | 5% | 13% | 12% | 9% |
| 846 | Västra Götaland | 0.8 | 0.8 | 4% | 6% | 10% | 16% | 12% |
| 847 | Västra Götaland | 0.8 | 0.8 | 12% | 6% | 13% | 10% | 4% |
| 848 | Västra Götaland | 0.8 | 0.8 | 4% | 8% | 13% | 12% | 10% |
| 849 | Västra Götaland | 0.8 | 0.8 | 10% | 5% | 15% | 10% | 4% |
| 850 | Västra Götaland | 0.8 | 0.8 | 13% | 5% | 8% | 11% | 7% |
| 851 | Västra Götaland | 0.8 | 0.8 | 8% | 6% | 8% | 12% | 10% |
| 852 | Västra Götaland | 0.8 | 0.8 | 6% | 7% | 14% | 10% | 10% |
| 853 | Västra Götaland | 0.8 | 0.8 | 10% | 5% | 7% | 12% | 11% |
| 854 | Västra Götaland | 0.8 | 0.8 | 10% | 5% | 11% | 10% | 8% |
| 855 | Västra Götaland | 0.8 | 0.8 | 9% | 5% | 11% | 12% | 7% |
| 856 | Västra Götaland | 0.7 | 0.8 | 8% | 5% | 13% | 10% | 7% |
| 857 | Västra Götaland | 0.7 | 0.8 | 11% | 4% | 11% | 10% | 5% |
| 858 | Västra Götaland | 0.7 | 0.7 | 12% | 5% | 13% | 8% | 5% |
| 859 | Västra Götaland | 0.7 | 0.7 | 13% | 4% | 9% | 10% | 5% |
| 860 | Västra Götaland | 0.7 | 0.7 | 10% | 6% | 15% | 9% | 2% |
| 861 | Västra Götaland | 0.7 | 0.7 | 12% | 4% | 4% | 15% | 4% |
| 862 | Västra Götaland | 0.7 | 0.7 | 8% | 7% | 9% | 12% | 6% |
| 863 | Västra Götaland | 0.7 | 0.7 | 7% | 6% | 12% | 10% | 7% |
| 864 | Västra Götaland | 0.7 | 0.7 | 10% | 5% | 17% | 8% | 3% |
| 865 | Västra Götaland | 0.7 | 0.7 | 8% | 5% | 12% | 10% | 7% |
| 866 | Västra Götaland | 0.7 | 0.7 | 8% | 6% | 12% | 11% | 6% |
| 867 | Västra Götaland | 0.7 | 0.7 | 7% | 6% | 11% | 12% | 4% |
| 868 | Västra Götaland | 0.7 | 0.7 | 9% | 5% | 16% | 10% | 0% |
| 869 | Västra Götaland | 0.7 | 0.7 | 9% | 5% | 16% | 8% | 2% |
| 870 | Västra Götaland | 0.7 | 0.7 | 10% | 4% | 12% | 10% | 4% |
| 871 | Västra Götaland | 0.7 | 0.7 | 9% | 5% | 12% | 9% | 5% |
| 872 | Västra Götaland | 0.7 | 0.7 | 8% | 5% | 10% | 11% | 5% |
| 873 | Västra Götaland | 0.7 | 0.7 | 12% | 4% | 12% | 8% | 3% |
| 874 | Västra Götaland | 0.7 | 0.7 | 8% | 4% | 14% | 8% | 5% |
| 875 | Västra Götaland | 0.7 | 0.7 | 8% | 3% | 13% | 11% | 4% |
| 876 | Västra Götaland | 0.7 | 0.7 | 9% | 6% | 15% | 7% | 1% |
| 877 | Västra Götaland | 0.7 | 0.7 | 10% | 3% | 10% | 10% | 4% |
| 878 | Västra Götaland | 0.7 | 0.7 | 7% | 4% | 17% | 6% | 7% |
| 879 | Västra Götaland | 0.7 | 0.7 | 8% | 4% | 19% | 6% | 3% |
| 880 | Västra Götaland | 0.7 | 0.7 | 5% | 6% | 12% | 9% | 8% |
| 881 | Västra Götaland | 0.7 | 0.7 | 6% | 4% | 11% | 9% | 8% |
| 882 | Västra Götaland | 0.6 | 0.7 | 6% | 5% | 10% | 12% | 4% |
| 883 | Västra Götaland | 0.6 | 0.6 | 8% | 4% | 16% | 7% | 3% |
| 884 | Västra Götaland | 0.6 | 0.6 | 7% | 5% | 10% | 9% | 5% |
| 885 | Västra Götaland | 0.6 | 0.6 | 8% | 4% | 17% | 6% | 1% |
| 886 | Västra Götaland | 0.6 | 0.6 | 6% | 4% | 14% | 8% | 6% |
| 887 | Västra Götaland | 0.6 | 0.6 | 7% | 4% | 15% | 7% | 3% |
| 888 | Västra Götaland | 0.6 | 0.6 | 6% | 5% | 8% | 10% | 6% |
| 889 | Västra Götaland | 0.6 | 0.6 | 6% | 4% | 12% | 8% | 6% |
| 890 | Västra Götaland | 0.6 | 0.6 | 5% | 5% | 14% | 7% | 5% |
| 891 | Västra Götaland | 0.6 | 0.6 | 6% | 4% | 14% | 7% | 4% |
| 892 | Västra Götaland | 0.6 | 0.6 | 5% | 4% | 12% | 8% | 6% |
| 893 | Västra Götaland | 0.6 | 0.6 | 5% | 4% | 18% | 5% | 3% |
| 894 | Västra Götaland | 0.6 | 0.6 | 7% | 3% | 7% | 9% | 8% |
| 895 | Västra Götaland | 0.6 | 0.6 | 6% | 3% | 14% | 7% | 3% |
| 896 | Västra Götaland | 0.6 | 0.6 | 7% | 4% | 13% | 6% | 4% |
| 897 | Västra Götaland | 0.6 | 0.6 | 4% | 3% | 13% | 8% | 5% |
| 898 | Västra Götaland | 0.5 | 0.5 | 6% | 3% | 15% | 4% | 3% |
| 899 | Västra Götaland | 0.5 | 0.5 | 7% | 3% | 10% | 7% | 3% |
| 900 | Västra Götaland | 0.5 | 0.5 | 4% | 3% | 14% | 7% | 4% |
| 901 | Västra Götaland | 0.5 | 0.5 | 4% | 5% | 6% | 9% | 6% |
| 902 | Västra Götaland | 0.5 | 0.5 | 3% | 4% | 11% | 8% | 5% |
| 903 | Västra Götaland | 0.5 | 0.5 | 1% | 5% | 10% | 9% | 5% |
| 904 | Västra Götaland | 0.5 | 0.5 | 6% | 3% | 13% | 5% | 2% |
| 905 | Västra Götaland | 0.5 | 0.5 | 2% | 5% | 10% | 8% | 5% |
| 906 | Västra Götaland | 0.5 | 0.5 | 1% | 5% | 11% | 6% | 5% |
| 907 | Västra Götaland | 0.5 | 0.5 | 5% | 3% | 10% | 6% | 2% |
| 908 | Örebro | 3.4 | 2.6 | 70% | 15% | 13% | 53% | 31% |
| 909 | Örebro | 2.6 | 2.0 | 56% | 10% | 16% | 41% | 19% |
| 910 | Örebro | 2.2 | 1.7 | 41% | 11% | 15% | 36% | 18% |
| 911 | Örebro | 2.1 | 1.6 | 41% | 11% | 13% | 33% | 17% |
| 912 | Örebro | 1.7 | 1.3 | 33% | 4% | 10% | 29% | 20% |
| 913 | Örebro | 1.7 | 1.3 | 28% | 10% | 8% | 30% | 19% |
| 914 | Örebro | 1.7 | 1.3 | 32% | 6% | 6% | 29% | 17% |
| 915 | Örebro | 1.5 | 1.2 | 28% | 8% | 6% | 24% | 17% |
| 916 | Örebro | 1.5 | 1.1 | 25% | 6% | 11% | 27% | 13% |
| 917 | Örebro | 1.5 | 1.1 | 22% | 10% | 8% | 29% | 13% |
| 918 | Örebro | 1.4 | 1.1 | 22% | 11% | 15% | 23% | 10% |
| 919 | Örebro | 1.4 | 1.1 | 25% | 7% | 14% | 22% | 10% |
| 920 | Örebro | 1.4 | 1.1 | 20% | 8% | 6% | 28% | 15% |
| 921 | Örebro | 1.4 | 1.0 | 19% | 8% | 8% | 26% | 15% |
| 922 | Örebro | 1.3 | 1.0 | 22% | 7% | 8% | 23% | 13% |
| 923 | Örebro | 1.1 | 0.9 | 15% | 8% | 15% | 17% | 7% |
| 924 | Örebro | 1.1 | 0.8 | 17% | 8% | 9% | 17% | 10% |
| 925 | Örebro | 1.1 | 0.8 | 8% | 10% | 9% | 20% | 15% |
| 926 | Örebro | 1.0 | 0.8 | 16% | 6% | 12% | 16% | 10% |
| 927 | Örebro | 1.0 | 0.8 | 13% | 6% | 9% | 17% | 11% |
| 928 | Örebro | 1.0 | 0.7 | 14% | 6% | 18% | 12% | 6% |
| 929 | Örebro | 0.9 | 0.7 | 14% | 6% | 16% | 11% | 4% |
| 930 | Örebro | 0.8 | 0.6 | 10% | 7% | 8% | 13% | 10% |
| 931 | Örebro | 0.7 | 0.6 | 11% | 5% | 5% | 12% | 9% |
| 932 | Örebro | 0.7 | 0.5 | 8% | 3% | 16% | 8% | 3% |
| 933 | Örebro | 0.6 | 0.5 | 5% | 5% | 10% | 9% | 7% |
| 934 | Örebro | 0.6 | 0.5 | 6% | 4% | 14% | 8% | 3% |
| 935 | Örebro | 0.6 | 0.5 | 3% | 4% | 12% | 9% | 8% |
| 936 | Örebro | 0.6 | 0.5 | 7% | 3% | 14% | 6% | 3% |
| 937 | Östergötland | 3.5 | 3.7 | 76% | 13% | 14% | 55% | 33% |
| 938 | Östergötland | 3.2 | 3.4 | 60% | 13% | 24% | 53% | 27% |
| 939 | Östergötland | 2.8 | 2.9 | 65% | 13% | 16% | 33% | 22% |
| 940 | Östergötland | 2.7 | 2.8 | 51% | 11% | 16% | 45% | 24% |
| 941 | Östergötland | 2.0 | 2.1 | 47% | 8% | 16% | 24% | 16% |
| 942 | Östergötland | 1.9 | 2.0 | 35% | 9% | 19% | 27% | 13% |
| 943 | Östergötland | 1.6 | 1.7 | 29% | 9% | 6% | 29% | 17% |
| 944 | Östergötland | 1.4 | 1.5 | 26% | 7% | 21% | 16% | 10% |
| 945 | Östergötland | 1.4 | 1.5 | 22% | 7% | 20% | 20% | 9% |
| 946 | Östergötland | 1.4 | 1.4 | 24% | 7% | 7% | 25% | 13% |
| 947 | Östergötland | 1.2 | 1.3 | 18% | 7% | 16% | 20% | 9% |
| 948 | Östergötland | 1.2 | 1.3 | 19% | 7% | 10% | 22% | 10% |
| 949 | Östergötland | 1.2 | 1.3 | 20% | 5% | 13% | 18% | 12% |
| 950 | Östergötland | 1.2 | 1.2 | 18% | 6% | 6% | 22% | 13% |
| 951 | Östergötland | 1.1 | 1.2 | 22% | 5% | 12% | 17% | 9% |
| 952 | Östergötland | 1.1 | 1.2 | 18% | 2% | 22% | 14% | 5% |
| 953 | Östergötland | 1.1 | 1.1 | 12% | 4% | 28% | 14% | 6% |
| 954 | Östergötland | 1.1 | 1.1 | 14% | 5% | 18% | 17% | 9% |
| 955 | Östergötland | 1.1 | 1.1 | 16% | 6% | 21% | 13% | 6% |
| 956 | Östergötland | 1.1 | 1.1 | 14% | 5% | 8% | 21% | 12% |
| 957 | Östergötland | 1.0 | 1.1 | 20% | 4% | 13% | 12% | 6% |
| 958 | Östergötland | 1.0 | 1.0 | 13% | 6% | 10% | 15% | 11% |
| 959 | Östergötland | 0.9 | 1.0 | 15% | 6% | 17% | 11% | 4% |
| 960 | Östergötland | 0.9 | 1.0 | 14% | 5% | 9% | 14% | 11% |
| 961 | Östergötland | 0.9 | 0.9 | 16% | 5% | 11% | 12% | 6% |
| 962 | Östergötland | 0.9 | 0.9 | 13% | 4% | 19% | 10% | 5% |
| 963 | Östergötland | 0.9 | 0.9 | 11% | 6% | 12% | 13% | 8% |
| 964 | Östergötland | 0.8 | 0.9 | 9% | 4% | 11% | 14% | 10% |
| 965 | Östergötland | 0.8 | 0.9 | 12% | 5% | 9% | 11% | 10% |
| 966 | Östergötland | 0.8 | 0.8 | 10% | 5% | 14% | 11% | 5% |
| 967 | Östergötland | 0.8 | 0.8 | 11% | 4% | 16% | 8% | 5% |
| 968 | Östergötland | 0.8 | 0.8 | 5% | 6% | 12% | 11% | 11% |
| 969 | Östergötland | 0.7 | 0.7 | 5% | 5% | 12% | 11% | 8% |
| 970 | Östergötland | 0.6 | 0.7 | 9% | 4% | 14% | 7% | 3% |
| 971 | Östergötland | 0.6 | 0.7 | 6% | 4% | 14% | 8% | 6% |
| 972 | Östergötland | 0.6 | 0.6 | 10% | 3% | 11% | 7% | 4% |
| 973 | Östergötland | 0.6 | 0.6 | 8% | 2% | 12% | 7% | 4% |
| 974 | Östergötland | 0.6 | 0.6 | 4% | 3% | 18% | 5% | 5% |
| 975 | Östergötland | 0.5 | 0.6 | 2% | 3% | 10% | 9% | 8% |
| 976 | Östergötland | 0.5 | 0.6 | 1% | 5% | 11% | 8% | 7% |
| 977 | Östergötland | 0.5 | 0.5 | 6% | 1% | 6% | 8% | 5% |
| 978 | Östergötland | 0.5 | 0.5 | 5% | 3% | 11% | 5% | 3% |
| 979 | Östergötland | 0.4 | 0.5 | 2% | 4% | 9% | 6% | 6% |
| 980 | Östergötland | 0.4 | 0.4 | 2% | 5% | 10% | 5% | 4% |
| 981 | Östergötland | 0.4 | 0.4 | 3% | 3% | 12% | 4% | 3% |
